# Supplementary material for: The Lateral Metalation of Isoxazolo[3,4-d]pyridazinones towards Hit-to-Lead Development of Selective Positive Modulators of Metabotropic Glutamate Receptors
Source: Molecules. 2023 Sep 25;28(19):6800. doi: 10.3390/molecules28196800 (PMC10574779; doi:10.3390/molecules28196800)

molecules

MDPI

Article

The lateral metalation of isoxazolo[3,4-d]pyridazinones towards hit-to-lead development of selective positive modulators of metabotropic glutamate receptors

Christina A. Gates [1], Donald S. Backos,[2] Philip Reigan,[2] and Nicholas R. Natale [1,*]

[1] University of Montana, Department of Biomedical and Pharmaceutical Sciences, 32 Campus Drive, Missoula MT 59812; christina.gates@umconnect.umt.edu

[2] Skaggs School of Pharmacy and Pharmaceutical Sciences, Anschutz Medical Campus, University of Colorado Denver, Aurora, CO 80045, United States; philip.reigan@cuanschutz.edu

* Correspondence: nicholas.natale@umontana.edu; Tel.: (406)-243-4132

Table of contents

1. General Experimental and larger version of **Scheme S1**…pp.1.2
2. Characterization of **3a, 3c, 3d, 3f, 3g, 3h, 4d and 4e**…pp. 3-17
3. **Table S1**. 4-phenyl series of [3,4-d] analogs…pp.18-19
4. Characterization of **2j** through **2n**….pp. 19-31

General Experimental

Starting materials and were prepared according to dal Piaz, The isoxazole acetal was prepared as we have previously described (Zhou, 1998; and Burkhart, 2001)

The regiochemistry of the [3,4-d]metalation was established by single crystal x-ray diffractometry (Campana, 2013)

Tetrahydrofuran (THF) was dried over activated sieves then distilled from sodium and benzophenone. Argon gas was passed through tubes with indicator Drierite for reactions which required an inert atmosphere. NMR spectra were recorded at 400 MHz, unless otherwise specified, in $CDCl_3$ solution and are reported in ppm. The mass spectra were obtained using chemical ionization unless otherwise noted and are reported as m/Z (relative intensity). Starting materials for the lateral metalation was prepared via Dal Piaz's method for 4-phenyl and 4-methyl 3,4-ds (citation).

All steps were performed under inert atmosphere unless otherwise noted. To predried round bottom cooled under argon the 3,4-d was added. After which dry THF was added in sufficient amount to reach a concentration of 50mM. The reaction was then placed and stirred in a cooling bath at the desired reaction temperature or based on solubility for 5min. Then 1 or 2 eq of the amine base was added via syringe dropwise over 5 min. The reaction mixture was then allowed to react for 30min, during which time the solution is usually observed to darken. Then add 1 or 2eq of a 1.7M solution of the electrophile in dry THF that has been cooled to 0°C or -78°C (done for 3,5-Cl_2) dropwise slowly. Allow to warm to room temperature, adding saturated ammonium chloride at about -20°C and allow to finish warming to room temperature. Concentration by rotary evaporator, dissolution in dichloromethane (DCM) and washed with water and brine was performed. The DCM layer was dried over sodium sulfate overnight. The Solution was then filtered and concentrated by rotary evaporator ,and purified by either PTLC or column with 6:1:1 hexanes, ethyl acetate, DCM.

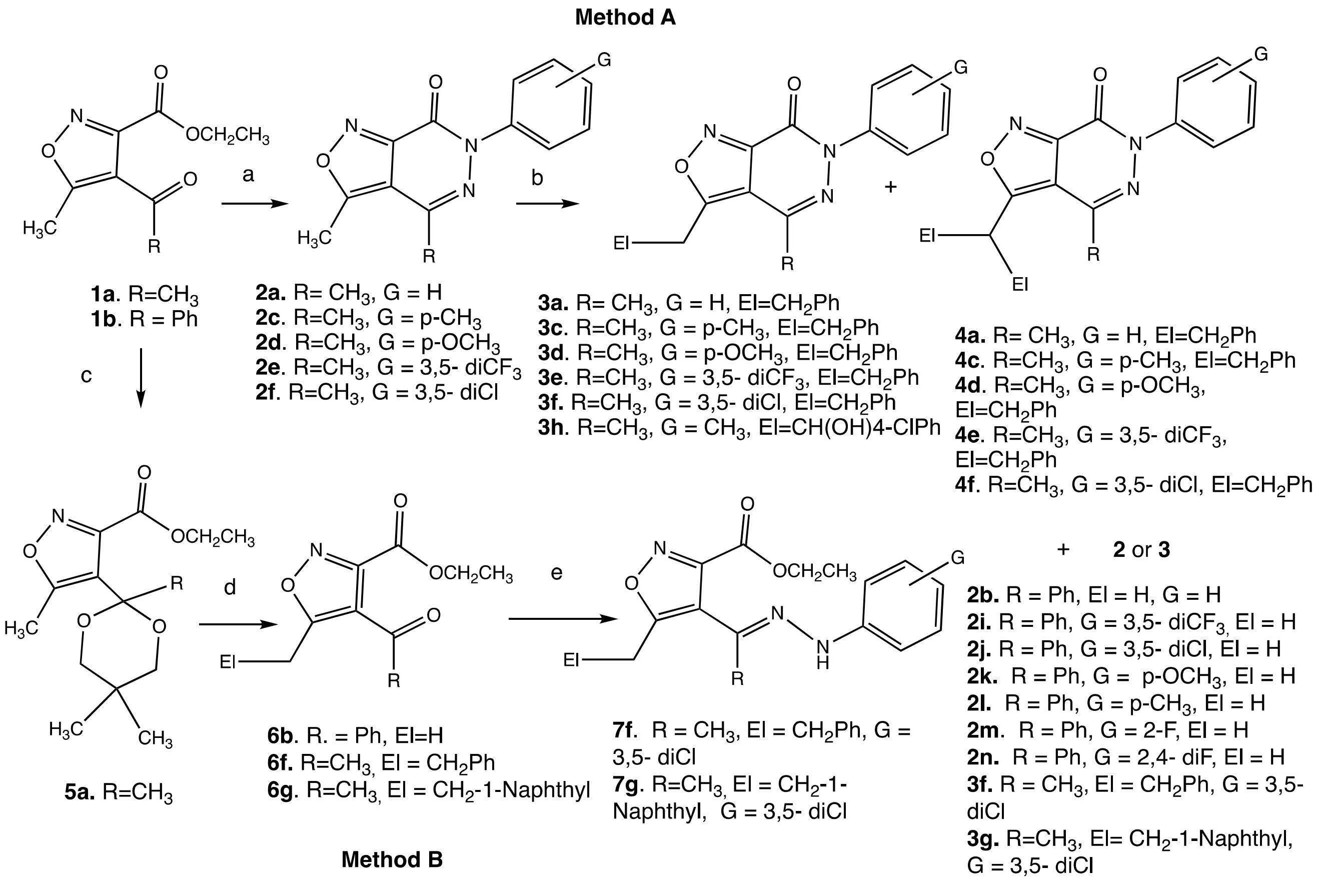

Scheme S1. Larger version of the Scheme

3-Phenylethyl, 4-methyl,6-phenylisoxazolo[3,4-*d*]pyridazin-7(6*H*)-one, 3a.

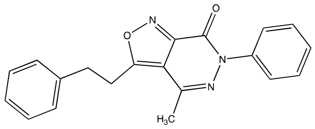

.

A. ^{1}H NMR

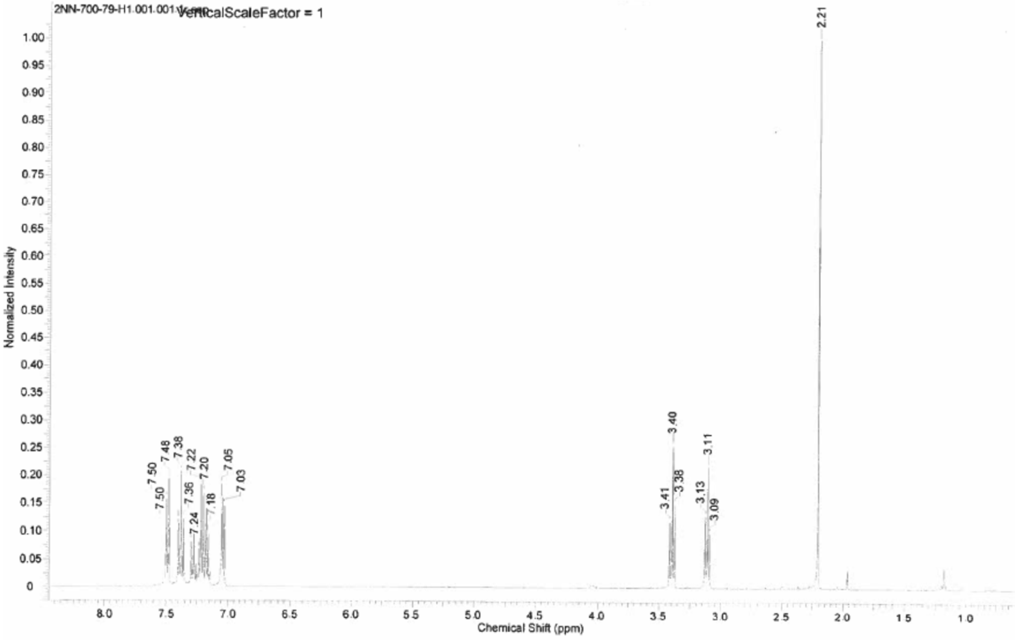

C. ^{13}C NMR

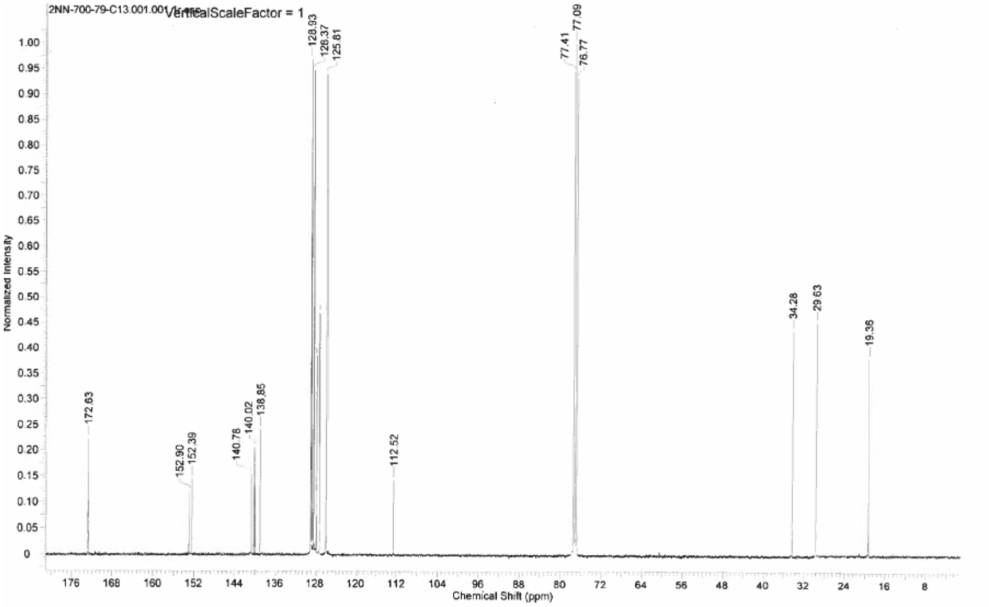

D. ^{13}C NMR zoom

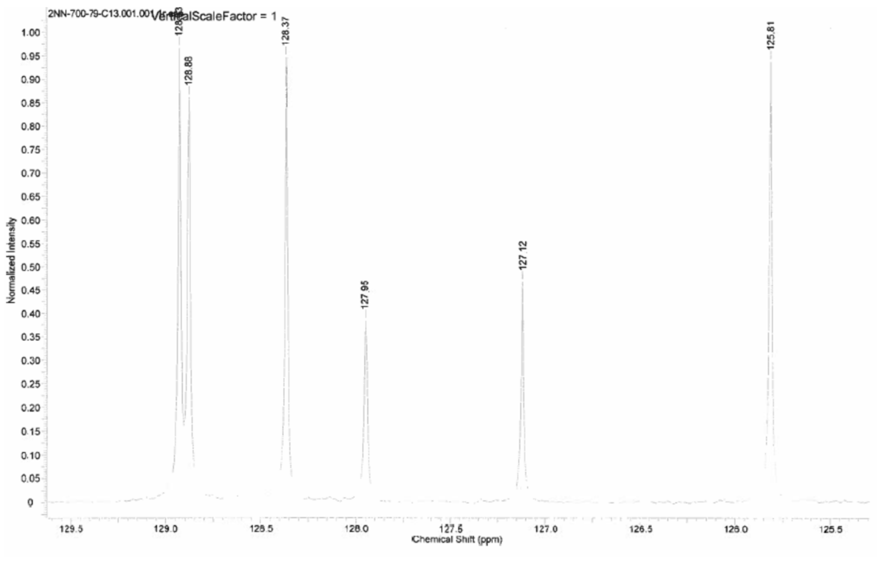

3-(2-(4-chlorophenyl)-2-hydroxyethyl)-4-methyl-6-(p-tolyl)isoxazolo[3,4-d]pyridazin-7(6H)-one. **3h.**

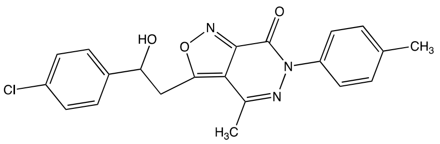

TLC (SiO_2 4:4:1 hexane-EtOAc-DCM) R_f 0.16. ^{1}H NMR (500 MHz, $CDCl_3$): δ 7.42 (d, J = 8.5 Hz, 2H); 7.259 (m, 4H); 7.08 (d, J=8.5 Hz, 2H); 5.1 (br. m., 1H); 4.04 (dd, J= 7Hz); 3.69 (dd, J = 15, 7Hz, 1H); 3.62 (dd, J= 7, 15 Hz); 2.40 (s, 3H); 2.386 (s, 3H). ^{13}C NMR: 169.51, 152.46, 138.87, 138.11, 138.01, 137.90, 134.37, 129.63, 129.51, 128.18, 125.55, 113.12, 43.19, 33.30, 21.13, 19.58. $C_{21}H_{18}ClN_3O_3$ MW 395.1; ESI-MS m/z 378.0079 (^{35}Cl, M-OH^+, 47% rel. I.), 379.9991 (^{37}Cl, M-OH^+, 15% rel. I.).

A. ^{1}H NMR

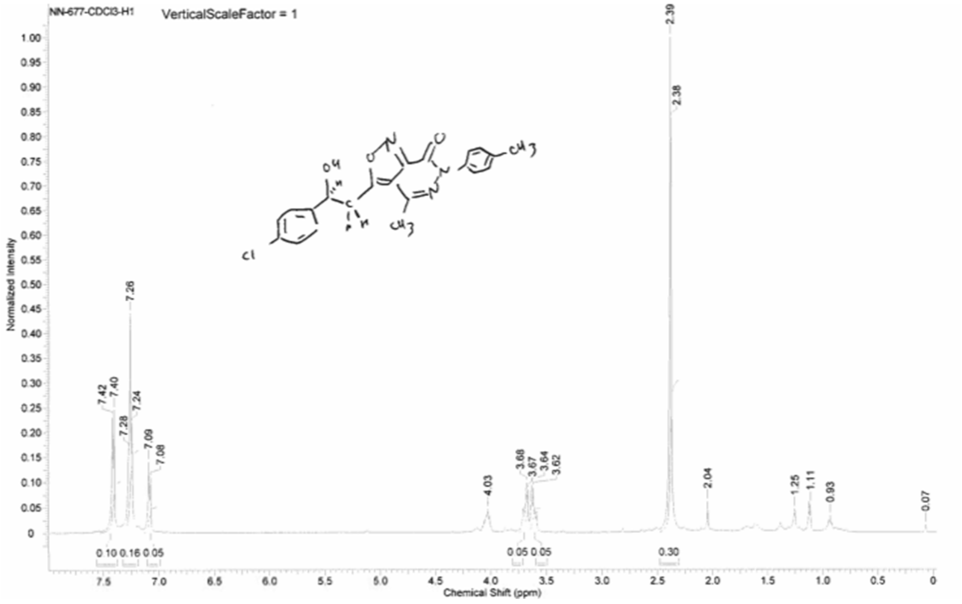

B. Expansion of ^{1}H NMR of hydroxy ethylene moiety of **3h.**

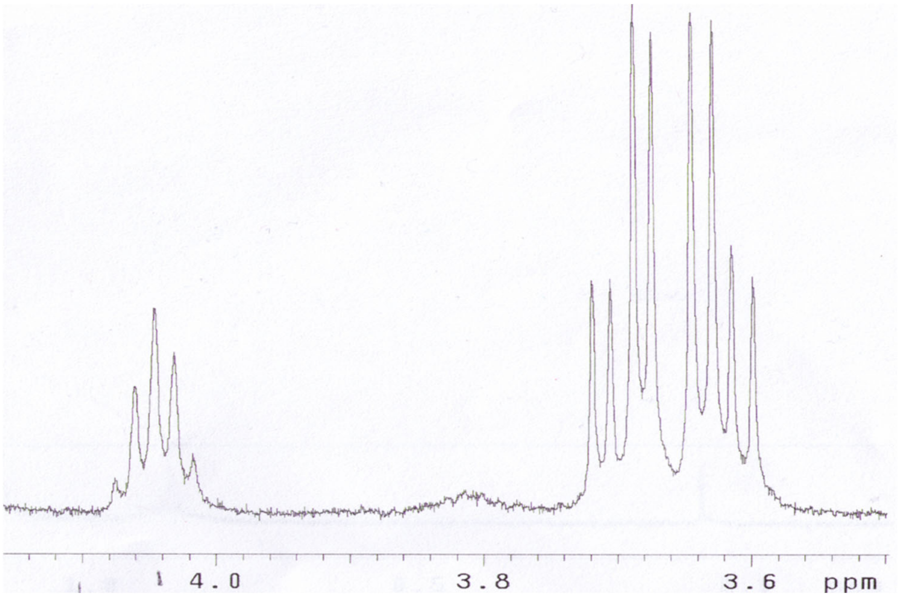

C. ^{13}C NMR of **3.h.**

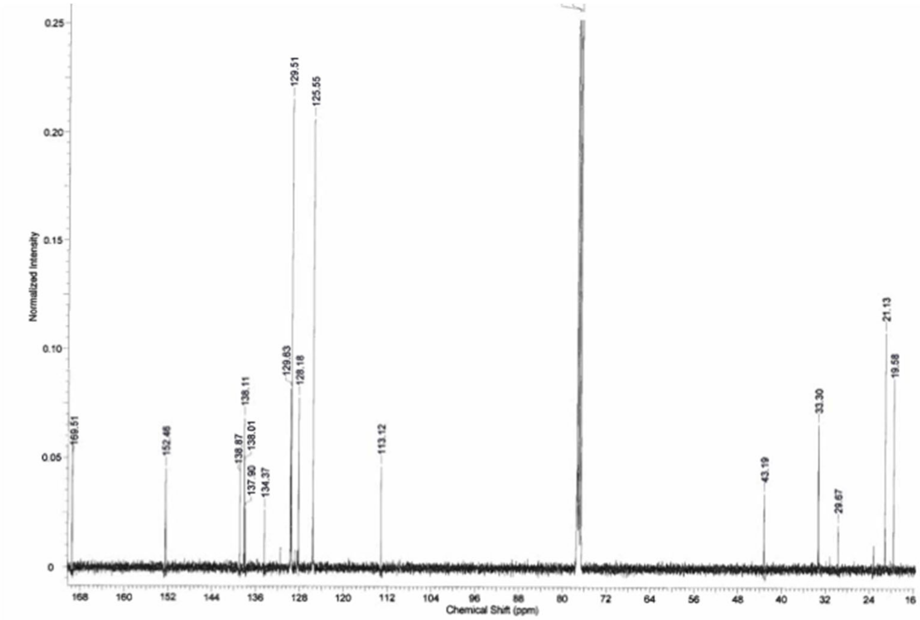

3. **4-methyl-3-phenethyl-6-(p-tolyl)isoxazolo[3,4-d]pyridazin-7(6H)-one 3c.**

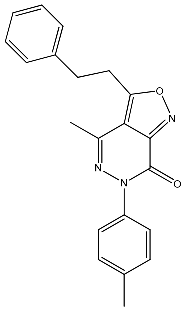

1H NMR (400 MHz, CDCl3): δ 7.29 (d, J=8 Hz, 2H); 7.12 (m, 5H); 6.97 (d, J=8Hz, 2H); 3.32 (t, J=8Hz, 2H); 3.03 (t, J=8Hz, 2H); 2.24 (s, 3H); 2.14 (s, 3H). 13C NMR: 172.44, 152.86, 152.35, 139.73, 138.79, 138.23, 137.91, 129.46, 128.88, 128.30, 127.06, 125.58, 112.49, 34.25, 29.58, 21.13, 19.30. C21H19N3O2 MW 345.39; ESI-MS m/z 346.1176 (M+1+, 100% rel. I.). HRMS: calc'd for C21H20N3O2 (M+H+): 346.1556, found: 346.1558. 0.6 ppm.

A. ^{1}H NMR

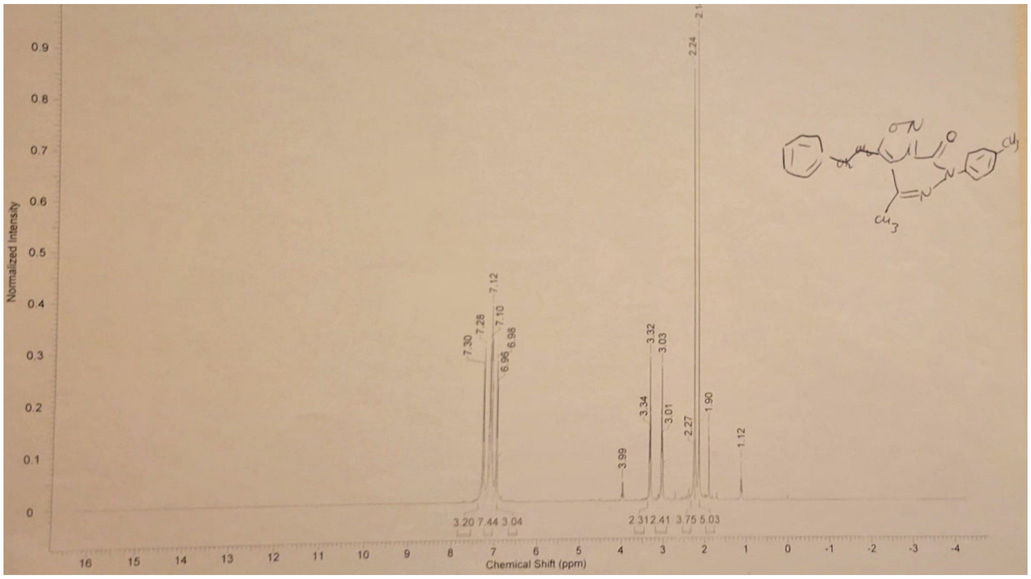

C. ^{13}C NMR

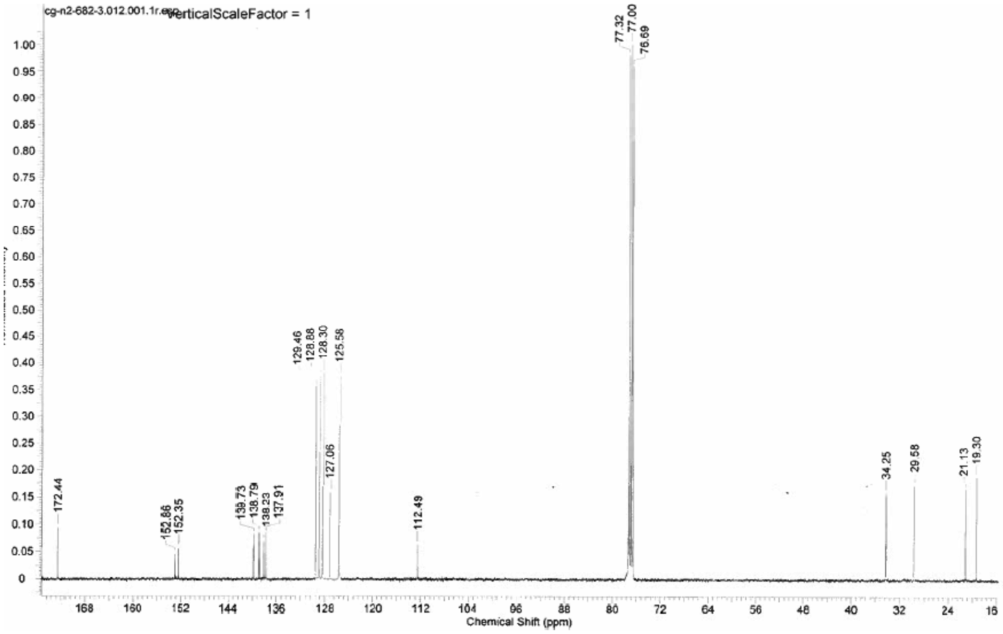

D. ^{13}C NMR zoom

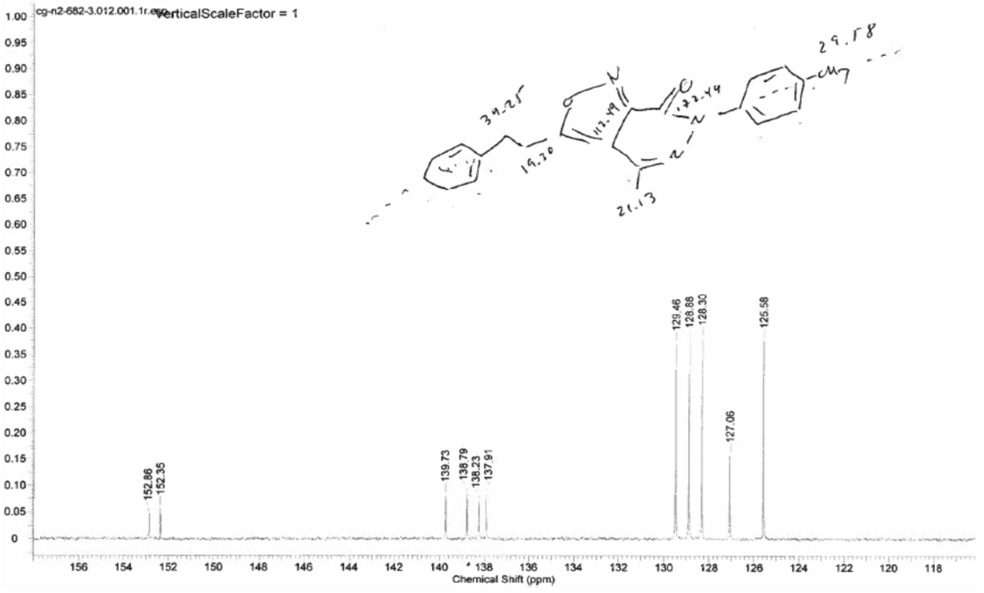

4. **6-(p-methoxyphenyl)-4-methyl-3-phenethyl-isoxazolo[3,4-d]pyridazin-7(6H)-one, 3d.**

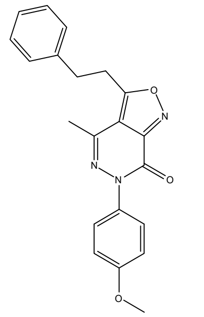

3.d. 1H NMR (400 MHz, CDCl3): δ 7.385 (d, J = 8Hz, 2H); 7.16-7.22 (m, 3H); 7.7.65 (d, J+8Hz, d); 6.89 (d, J = 8Hz, 2H); 3.73 (s, 3H); 3.38 (t, J=8Hz, 2H); 3.11 (t, J=8Hz, 2H); 2.20 (s, 3H). 13C NMR: 172.57, 159.03, 152.95, 152.37, 139.80, 138.86, 133.77, 128.91, 127.05,114.13, 55.58, 34.27, 29.62, 19.35. C21H19N3O3 MW: 361.3. HRMS calc'd for C21H20N3O3 (M+H+): 362.1505, found: 362.1506. 0.3 ppm**.**

A. ¹H NMR

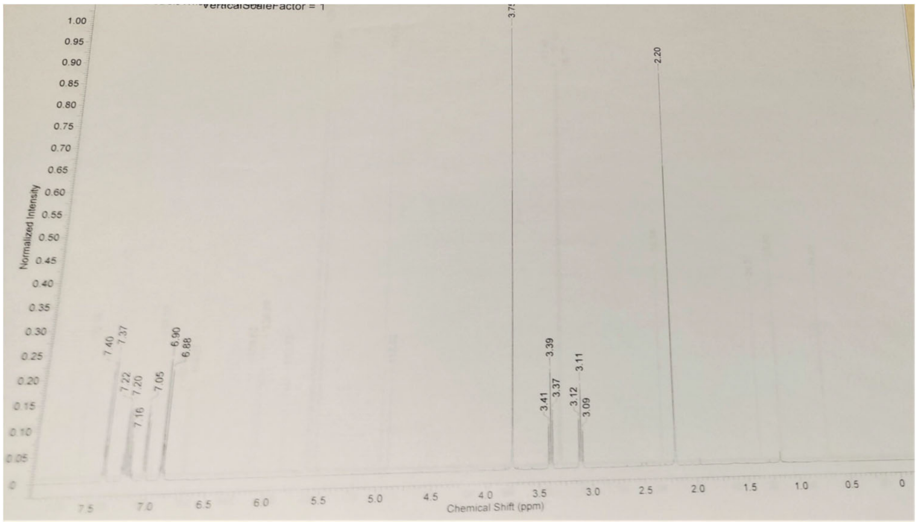

B. ^{13}***C NMR***

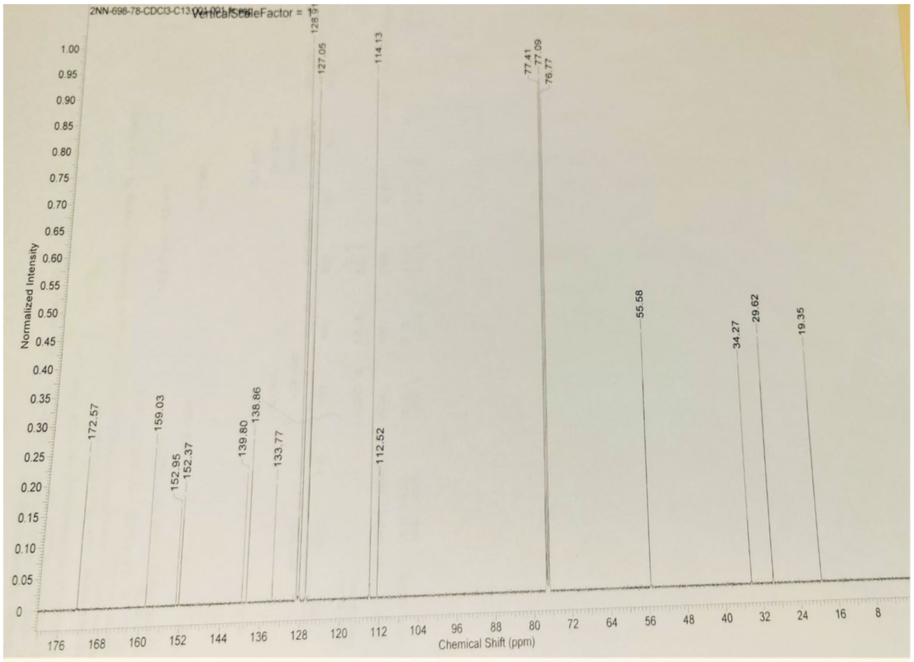

6. **6-(p-methoxyphenyl)-3-(1,3-diphenylpropan-2-yl)-4-methyl-isoxazolo[3,4-d]pyridazin-7(6H)-one, 4d.**

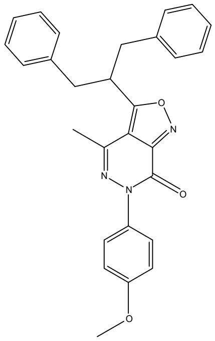

4.d. 1H NMR (400 MHz, CDCl3): δ 7.41 (d, J=8Hz, 2H); 7.19-7.25 (m, 6H); 7.19 (d, J=8Hz, 4H); 6.94 (d, J=8Hz, 2H); 3.83 (s, 3H); 3.81 (pentet, J = 8Hz, 1H); 3.28 (d, J=8Hz, 4H); 1.85 (s, 3H). 13C NMR: 174.46, 158.96, 152.91, 151.94, 139.62, 138.03, 133.65, 128.82, 128.59, 126.93, 114.06, 113.43, 55.55, 45.01, 40.82, 18.96. C28H25N3O3 MW: 451.5. HRMS calc'd for C28H26N3O3 (M+H+): 452.1974, Found: 452.1975. 0.2 ppm.

A. ^{1}**H NMR**

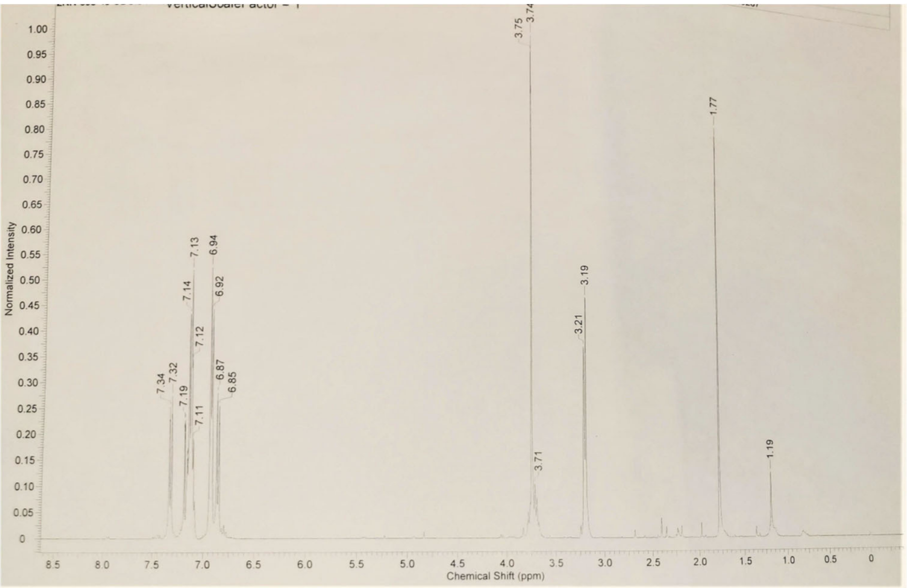

B. ^{13}C NMR

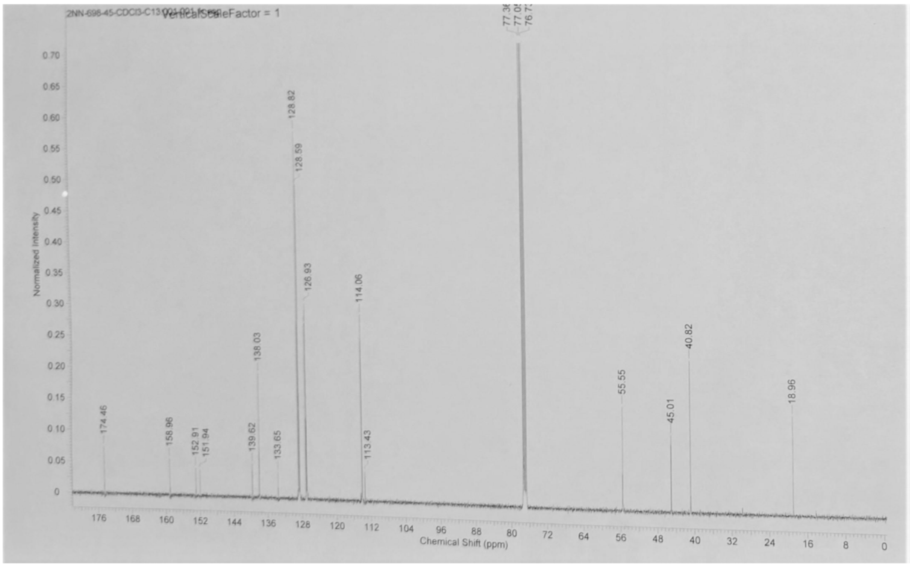

7. **6-(3,5-dichlorophenyl)-4-methyl-3-phenethylisoxazolo[3,4-d]pyridazin-7(6H)-one, 3f.**

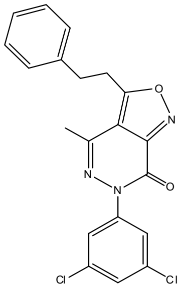

1H NMR (400 MHz, CDCl3): δ 7.515 (d, J = 4 Hz, 2H); 7.18-7.27 (m, 5H); 7.04 (d, 1H); 3.41 (t, 3 J = 8Hz, 2H); 3.12 (t, 3 J = 8Hz, 2H); 2.2 (s, 3H). 13C NMR: 173.01, 152.72; 151.99; 142.12; 140.88; 138.62; 134.81; 128.91; 128.28; 127.15; 124.20; 112.29; 34.20; 29.61; 19.26. C20H15Cl2N3O2 MW: 400.26; ESI-MS m/z 400 (M+H, 100%% rel. I.); 402 (M+H+2, 67.7); 404 (M+H+4, 12.2). HRMS Calc'd for C20H16Cl2N3O2 400.0620, Found: 400.0622. 0.5 ppm.

A. ^{1}H NMR

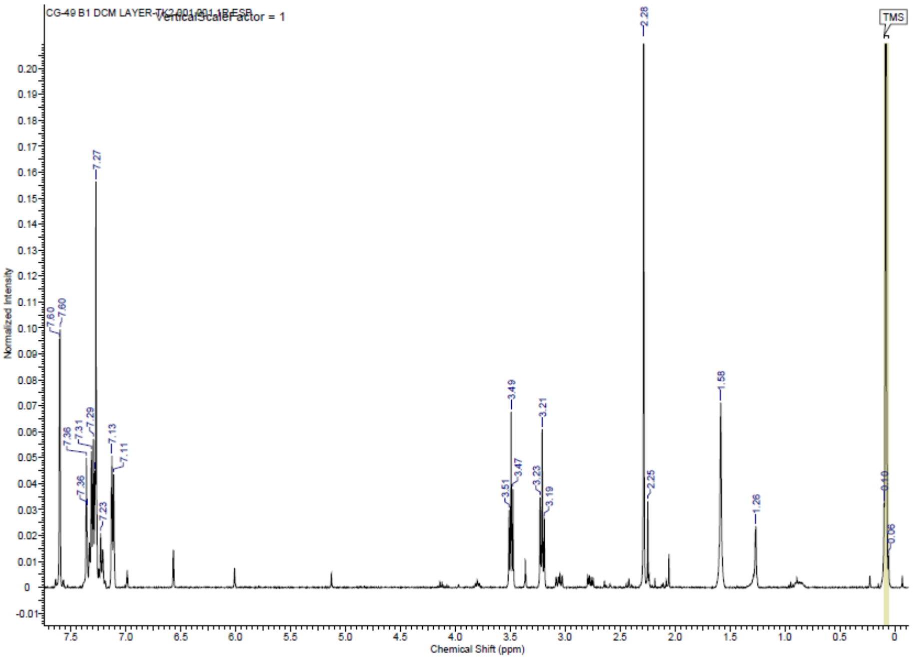

B. ^{1}H NMR Aromatic expanded

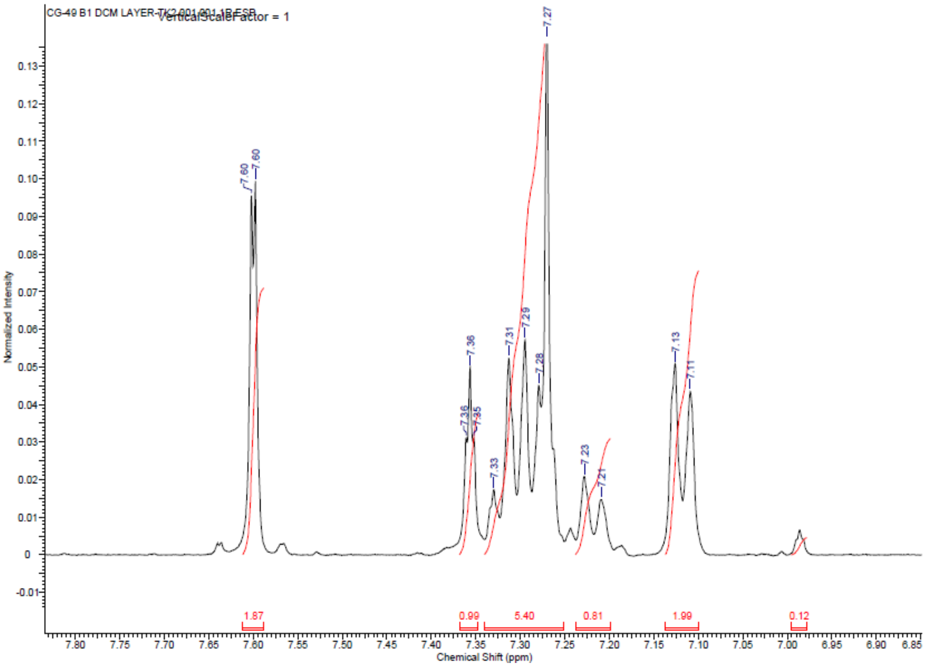

C. ^{13}C NMR

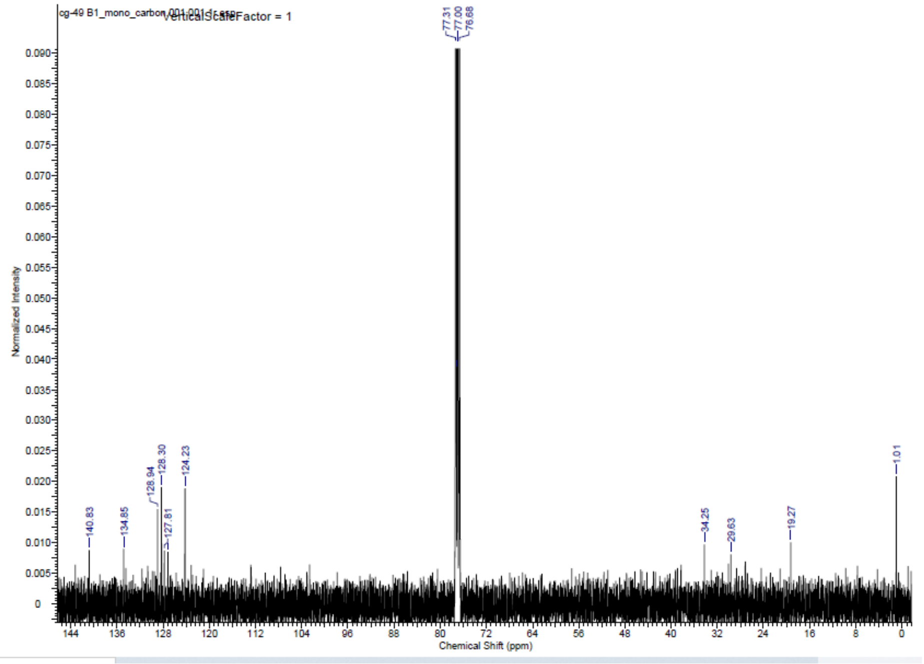

D. ^{13}C NMR zoom

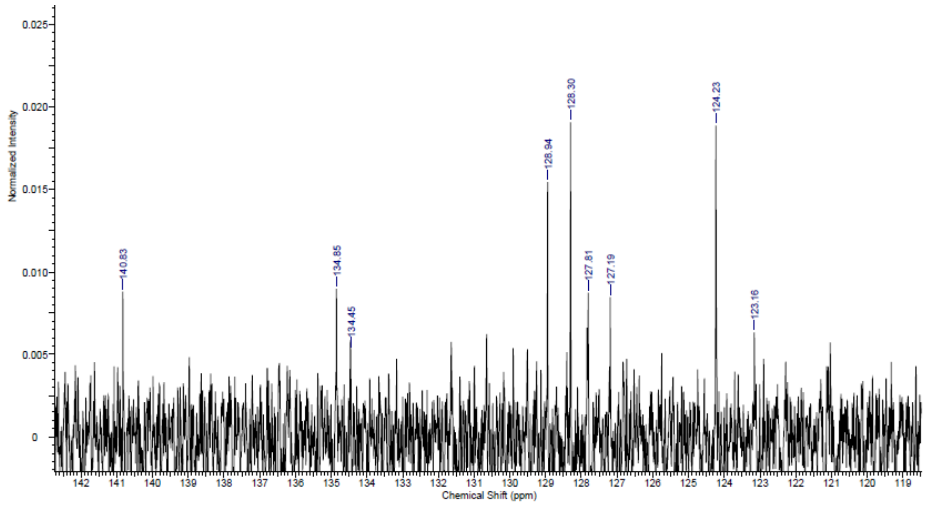

13. ***(E)-ethyl-4-(1-(2-(3,5-dichlorophenyl)hydrazono)ethyl)-5-phenethylisoxazole-3-carboxylate, open precursor to 4f.***

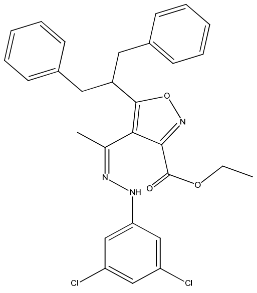

. 7.f. 1H NMR (400 MHz, CDCl3): δ 4.34 (q, J = 8 Hz, 2H); 3.17 (t, 2H); 2.98 (t, 2H); 1.80 (s, 3H); 1.32 (t, J = 8 Hz, 3H). C22H21Cl2N3O3 MW: 446.3; ESI-MS m/z 446 (M+1+, 100% rel. I.), 448 (M+3+, 67.4).

8. ***6-(3,5-dichlorophenyl)-4-methyl-3-(1,3-diphenylpropan-2-yl)-isoxazolo[3,4-d]pyridazin-7(6H)-one, 4f.***

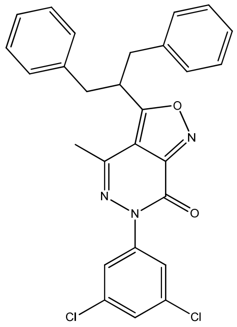

4.f. 1H NMR (400 MHz, CDCl3): δ 7.532 (d, J = 1.7 Hz, 2H); 7.32 (t, J=1.7(x2),1H); 7.25-7.19 (m, 6H); 7.01-6.99 (d, J = 6.4Hz, 4H); 3.81-3.77 (t, 3 J = 8Hz (x2), 1H); 3.30 (s, 2H); 3.28 (s,2H);1.85(s,3H);1.59(s,1H). 13C NMR: 174.99; 140.68; 137.87; 134.75; 128.84; 128.50; 127.16; 124.11; 45.15; 40.83; 18.88 C27H21Cl2N3O2 MW: 490.39; HRMS 489 (M-H, 100%% rel. I.); 491 (M+H). Calc'd for C27H21Cl2N3O2 490.3805, Found: 490.1226.

A. ^{1}H NMR

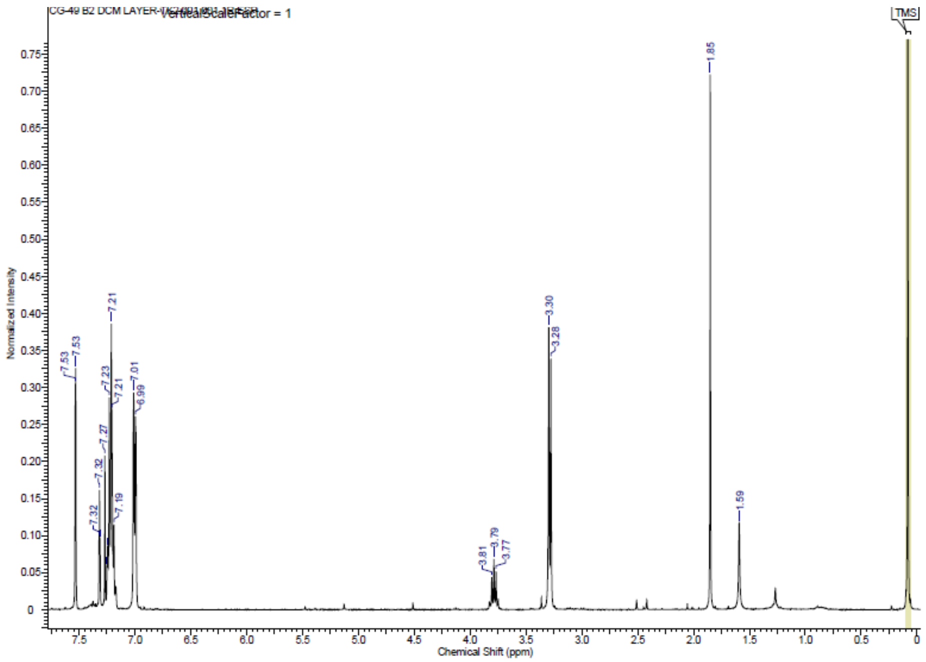

B. ^{1}H NMR Aromatic expanded

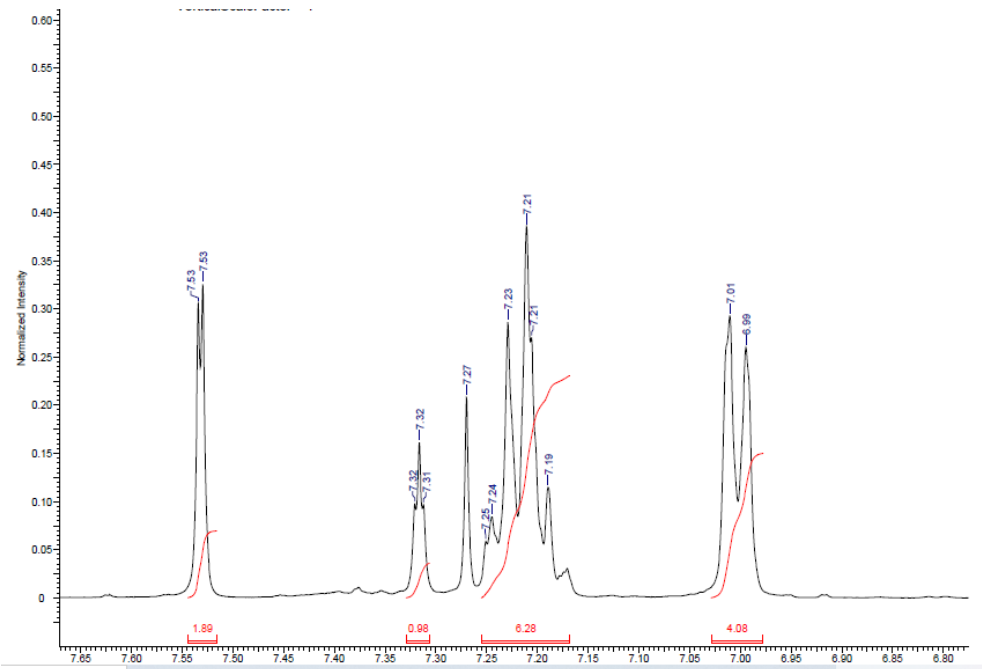

C. ^{13}C NMR

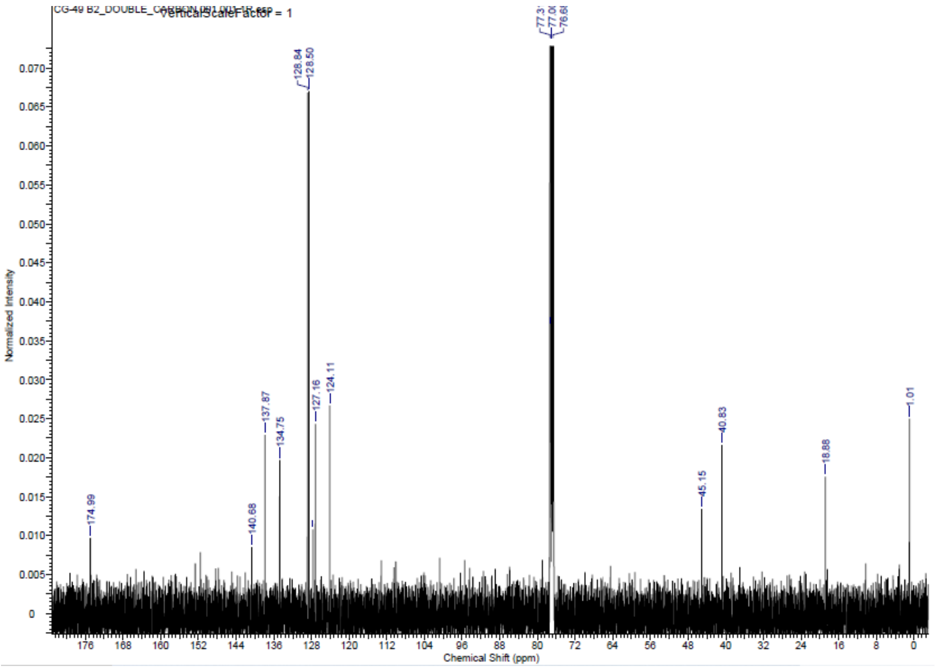

D. ^{13}C NMR zoom

10. ***6-(3,5-bistrifluoromethylphenyl)-4-methyl-3-(1,3-diphenylpropan-2-yl)-isoxazolo[3,4-d]pyridazin-7(6H)-one, 4e.***

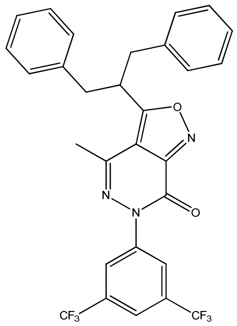

4.e. ^{1}H NMR (400 MHz, CDCl3): δ 8.13 (s, 2H);7.82(s, 1H); 7.04 (d, 1H); 7.25-7.18 (m, J = 8Hz, 6H); 7.02-7.00 (d, J = 8Hz, 4H); 3.82-3.79 (m,1H); 3.31 (s 2H); 3.29 (s,2H);1.88(s,3H); 1.6 (s,1H). 13C NMR 175.27,137.82,128.86,128.48,127.17,113.23,45.23,18.90, C29H21F6N3O2 MW: 557.49; HRMS m/z 558 (M+H, 100%% rel. I.); 559 (M+H+2); Calc'd for C29H21F6N3O2 557.49, Found: 557.1153.

A. ^{1}H NMR

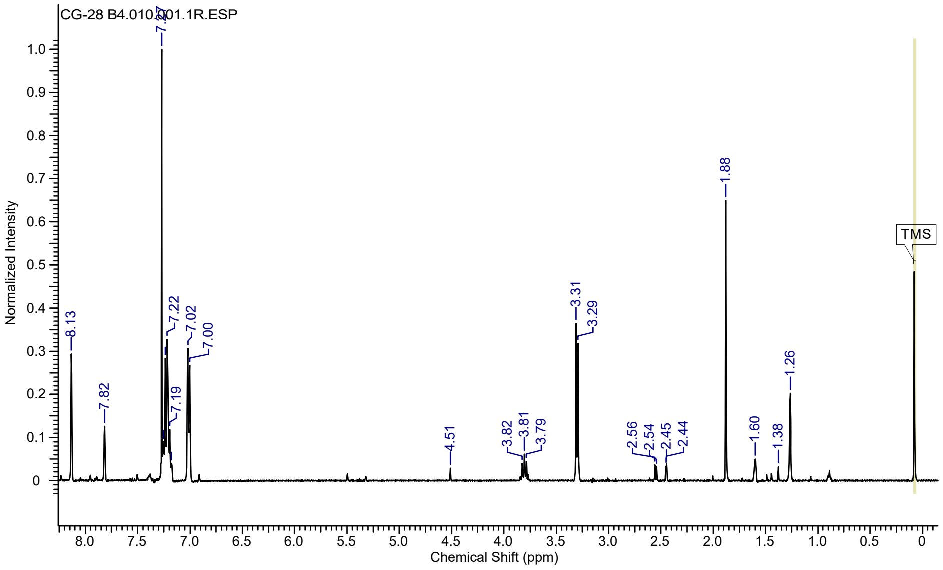

B. ^{1}H NMR Aromatic expanded

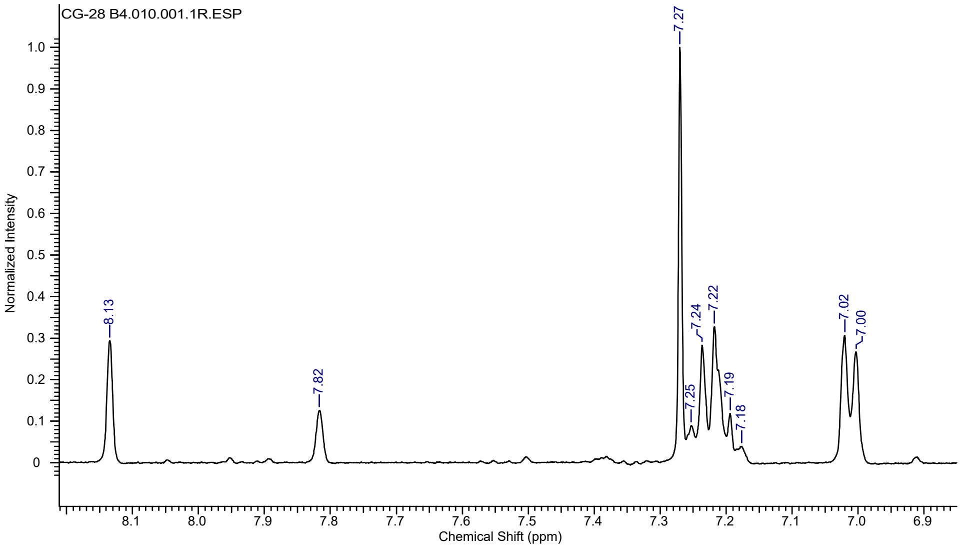

C. ^{13}C NMR

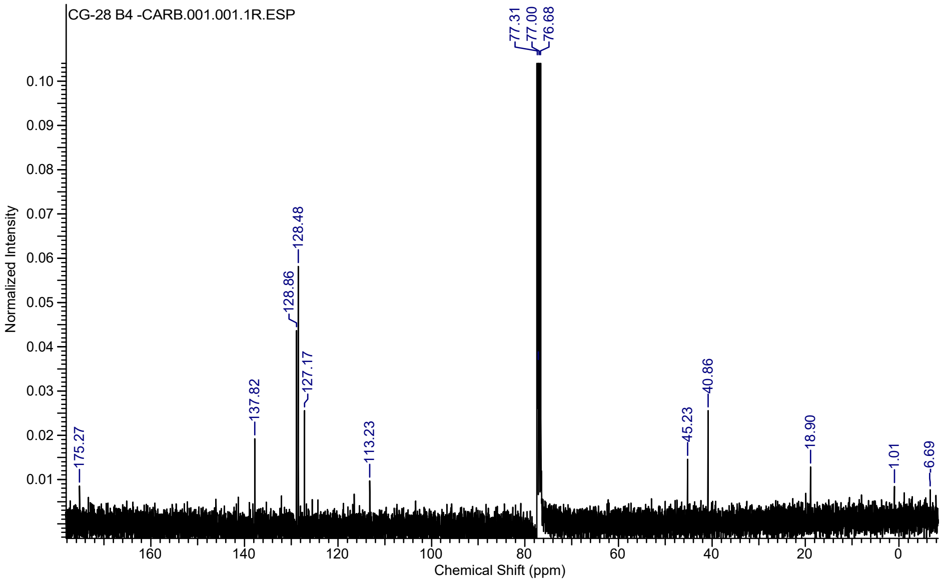

D. ^{13}C NMR zoom

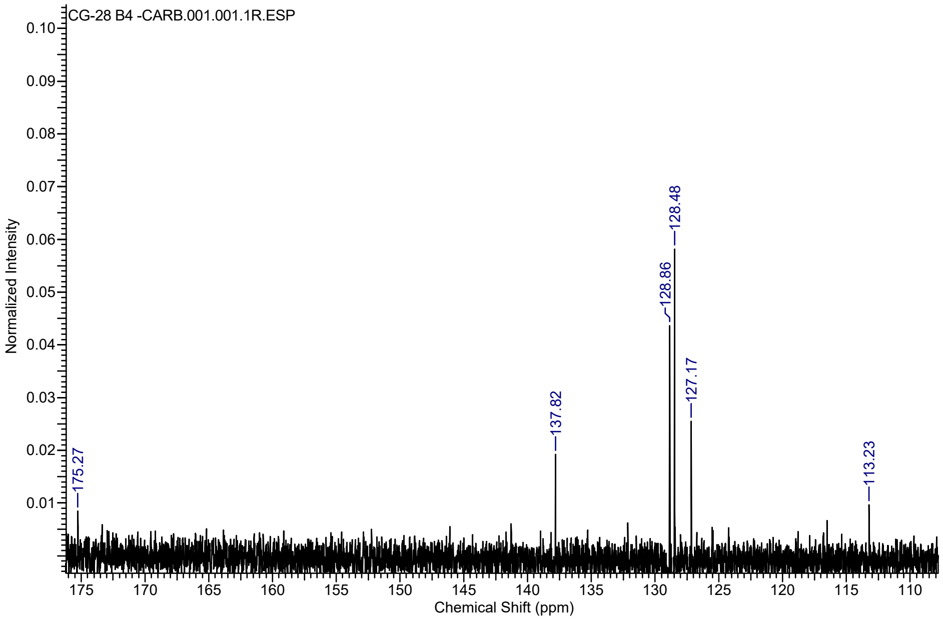

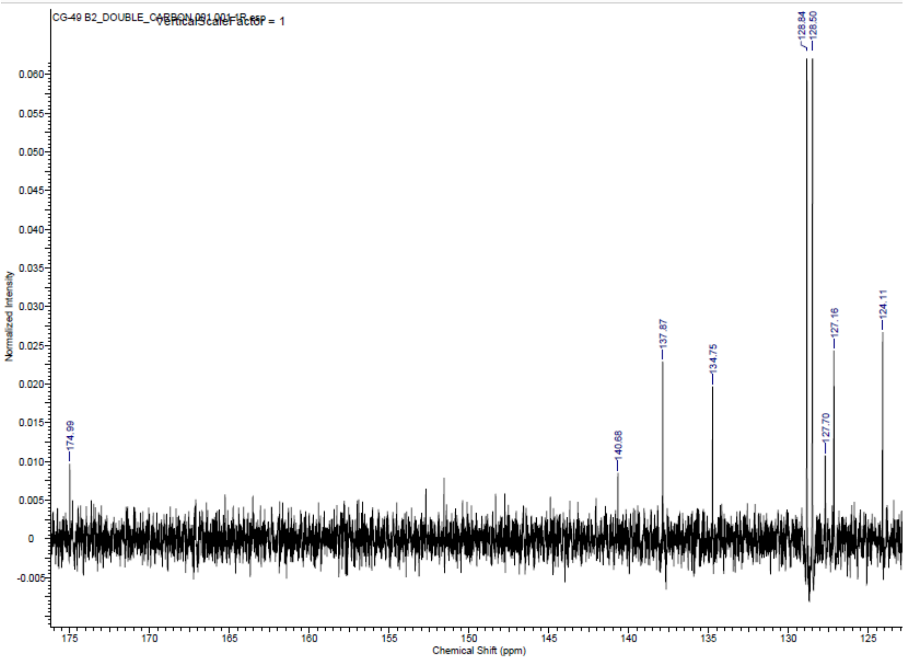

14. ***6-(3,5-dichlorophenyl)-4-methyl-3-(2-(naphthalen-1-yl)ethyl)isoxazolo[3,4-d]pyridazin-7(6H)-one, 3g.***

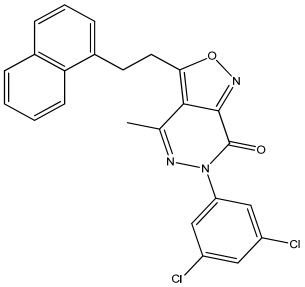

. 3.g. ^{1}H NMR (400 MHz, CDCl3): δ 7.96 (d, J = 8 Hz, 1H); 7.90 (d, J = 8 Hz, 1H); 7.79 (d, J = 8 Hz, 1H); 7.37-7.61 (m, H); 7.56 (d, J = 2Hz, 2H); 7.35 (t, 1H); 7.35 (d, J = 2Hz, 1H); 7.21 (d, J = 8 Hz, 1H), 3.675 (dd, 2H): 3.64 (dd, 2H); 2.00 (s, 3H). ^{13}C NMR: δ 172.98, 152.67, 152.0, 142.11, 140.73, 134.83, 134.57, 131.20; 129.26, 127.0, 126.65, 125.57. 124.22, 122.62, 112.46, 31.42, 28.65, 18.92. C24H17Cl2N3O2 MW: 450.32; ESI-MS m/z 450 (M+H, 100% rel. I.); 452 (M+H+2, 68.9); 452 (M+H+4, 13.1). HRMS Calc'd for C24H18Cl2N3O2 450.0776, Found: 450.0775. -0.2 ppm.

A. ^{1}H NMR

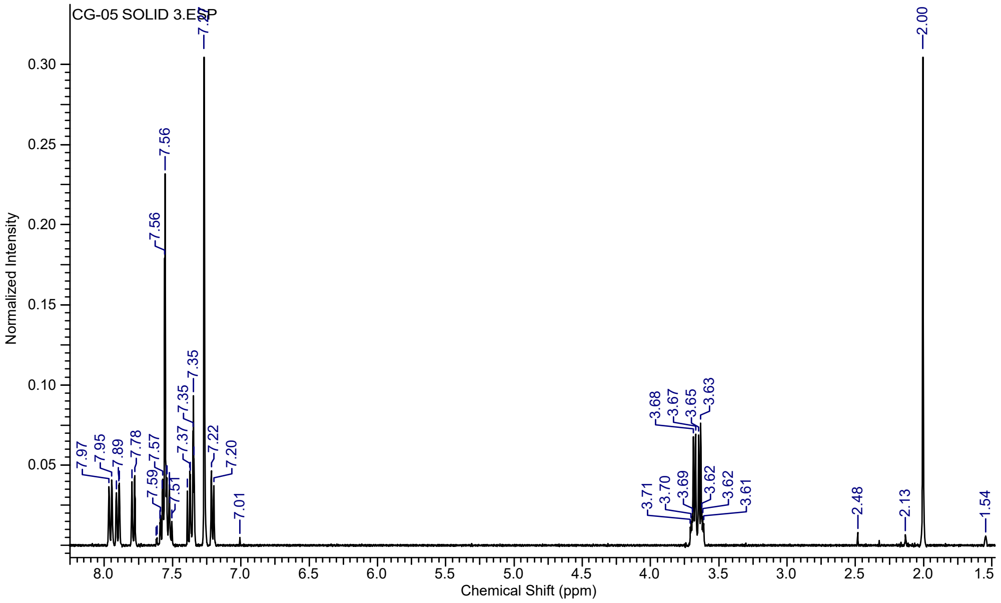

B. ^{1}H NMR Aromatic expanded

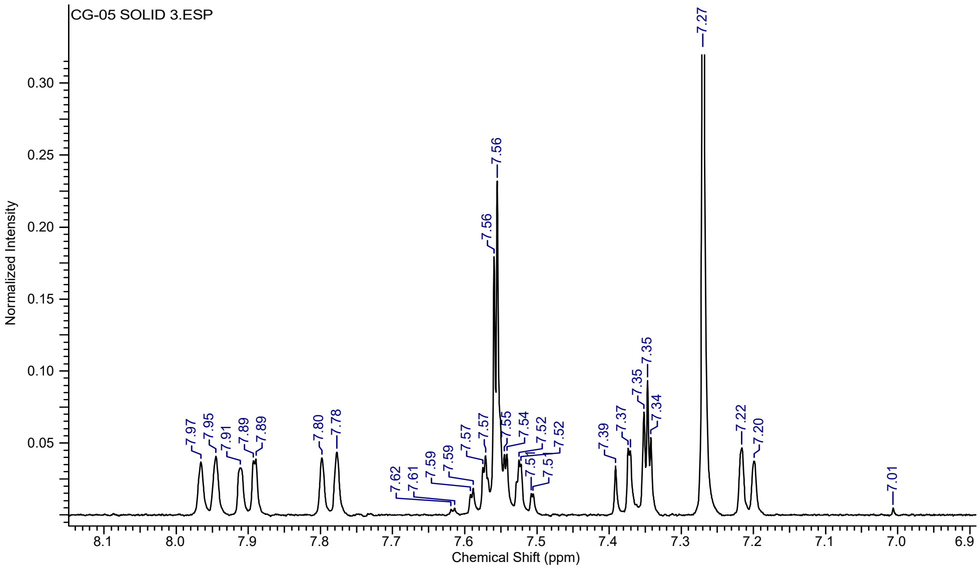

C. ^{13}C NMR

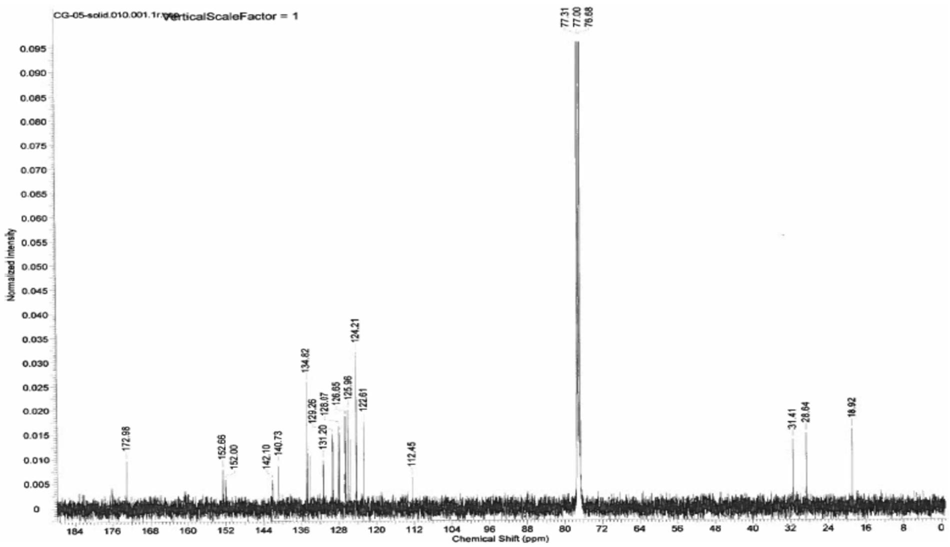
CG-05-solid.010.001.1r.esp
VerticalScaleFactor = 1
Normalized Intensity
Chemical Shift (ppm)
172.98
152.66
152.00
142.10
140.73
134.82
129.26
131.20
128.07
126.65
125.96
124.21
122.61
112.45
77.31
77.00
76.68
31.41
28.64
18.92

Table S1. 4-phenyl series of [3,4-d] analogs

**2j** cg-15;17;25;33;36	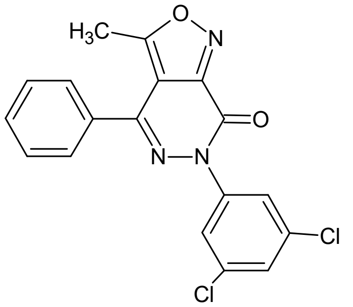	212.5 Molecular Formula = $C_{18}H_{11}Cl_2N_3O_2$ Formula Weight = 372.20484
**2i** cg-19;34;35	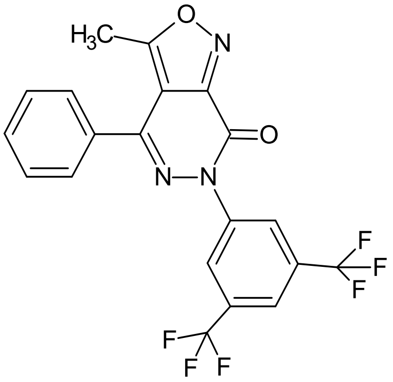	205 Molecular Formula = $C_{20}H_{11}F_6N_3O_2$ Formula Weight = 439.3106592
**2k** cg-22;24	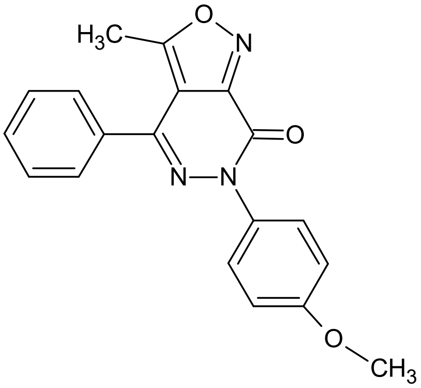	40 Molecular Formula = $C_{19}H_{15}N_3O_3$ Formula Weight = 333.3407
**2l** cg-32	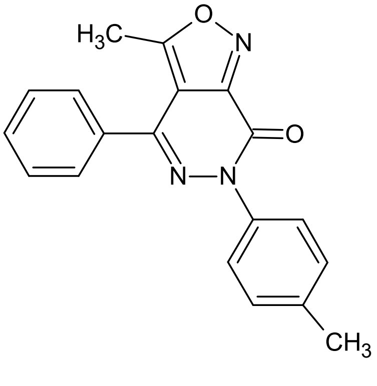	119 Molecular Formula = $C_{19}H_{15}N_3O_2$ Formula Weight = 317.3413

**2n**<br>cg-37	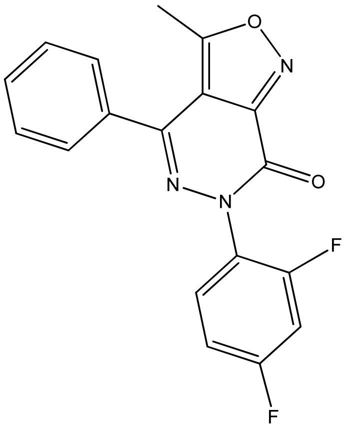	196<br><br>Chemical Formula: $C_{18}H_{11}F_2N_3O_2$<br>Molecular Weight: 339.30
**2m**<br>cg-38	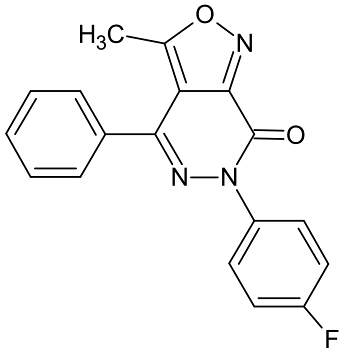	78.8<br><br>Molecular Formula = $C_{18}H_{12}FN_3O_2$<br>Formula Weight = 321.3051832

*9. **6-(3,5-dichlorophenyl)-3-methyl-4-phenylisoxazolo[3,4-d]pyridazin-7(6H)-one. 2j.***

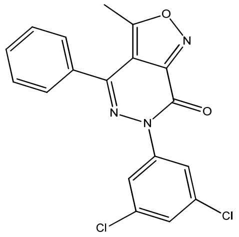

1H NMR (400 MHz, CDCl3): δ 7. 68 (2H); 7.57 (5H); 7.38 (1H); 5.59 (s, 3H). 13C NMR: δ 208.20,194.33,181.58,170.91,164.94 152.67, 152.0, 142.11, 140.73, 134.83, 134.57, 131.20; 129.26, 127.0, 126.65, 125.57. 124.22, 122.62, 112.46, 31.42, 28.65, 18.92.HRMS Calc'd for C18H1135Cl2N3O2+H 372.0307, Found: 372.0309. 0.5 ppm.

A. ^{1}H NMR

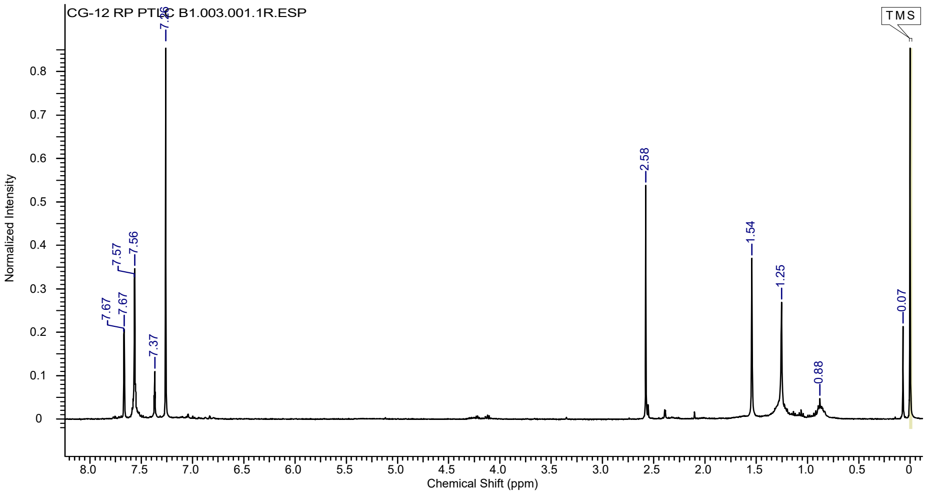

B. ^{1}H NMR Aromatic expanded

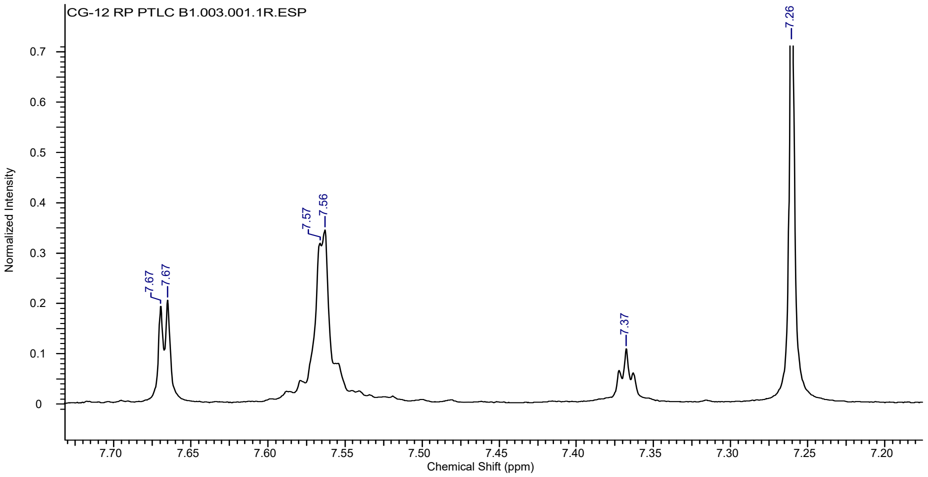

C. ^{13}C NMR

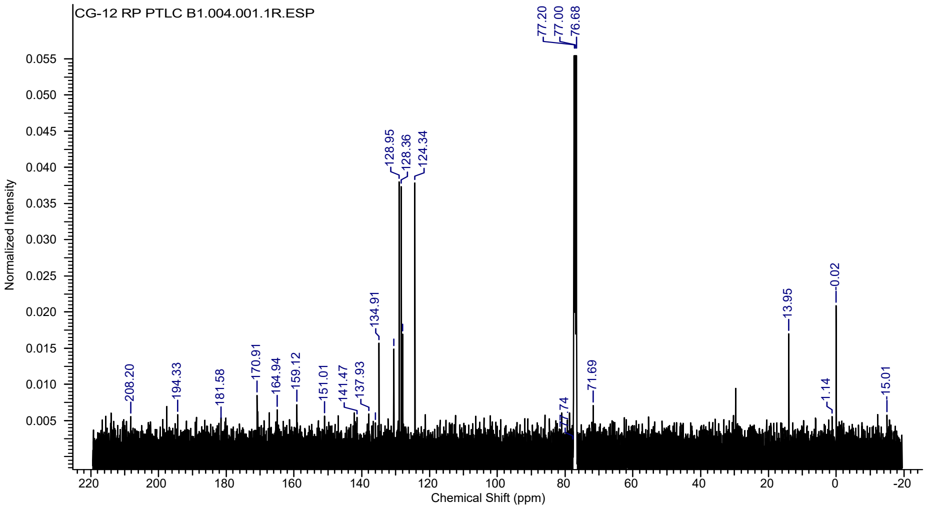

D. ^{13}C NMR zoom

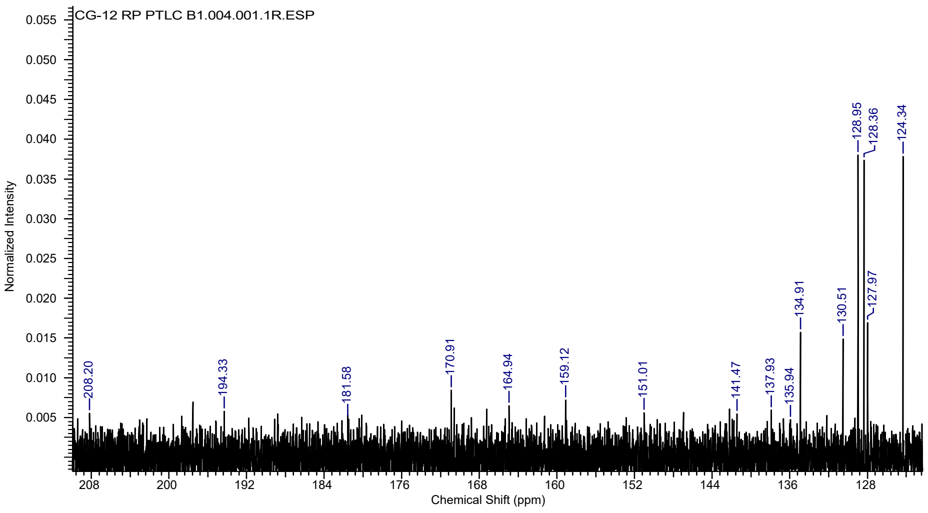

11. ***6-(4-methoxyphenyl)-3-methyl-4-phenyl-6H,7H-[1,2]oxazolo[3,4-d]pyridazin-7-one, 2k.***

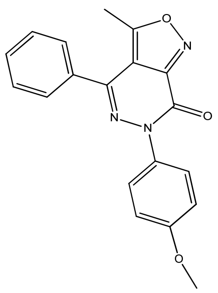

^{1}H NMR (400 MHz, CDCl3): δ 7.58-7.53 (t, J=8,7H);7.00-6.98(d, J=8,2H); 3.85(s,3H);2.58 (s, 3H).^{13}C NMR 170.38; 159.0; 152.82; 152.73; 142.63; 133.77; 130.11; 28.76; 128.40; 127.02; 114.03;111.37;55.52;13.92 C19H15N3O3 MW: 333.35; HRMS m/z: 333.1113 (100.0%), 334.1147 (20.5%), 335.1181 (2.0%), 334.1084 (1.1%); Calc'd for C19H15N3O3 333.3407, Found: 333.1113.

A. ^{1}H NMR

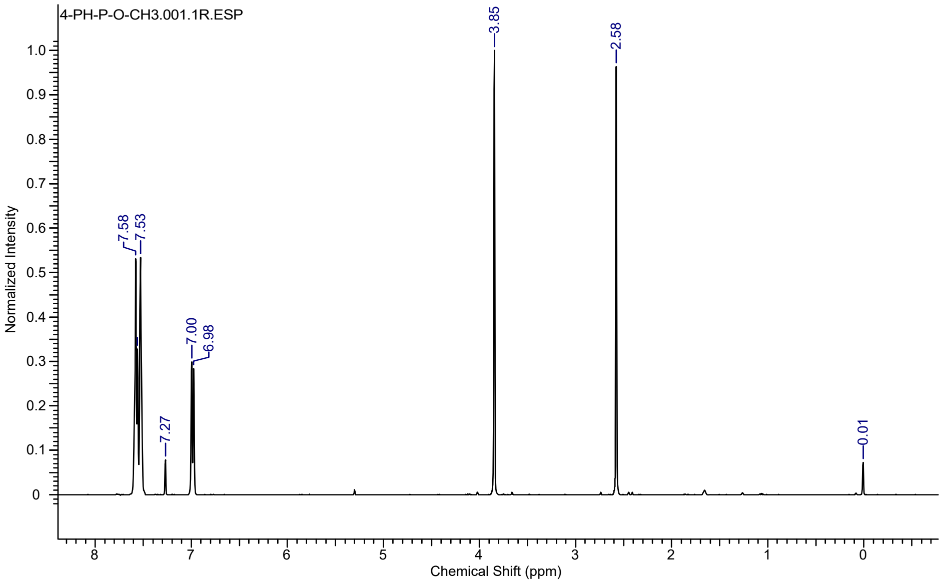

B. ^{1}H NMR Aromatic expanded

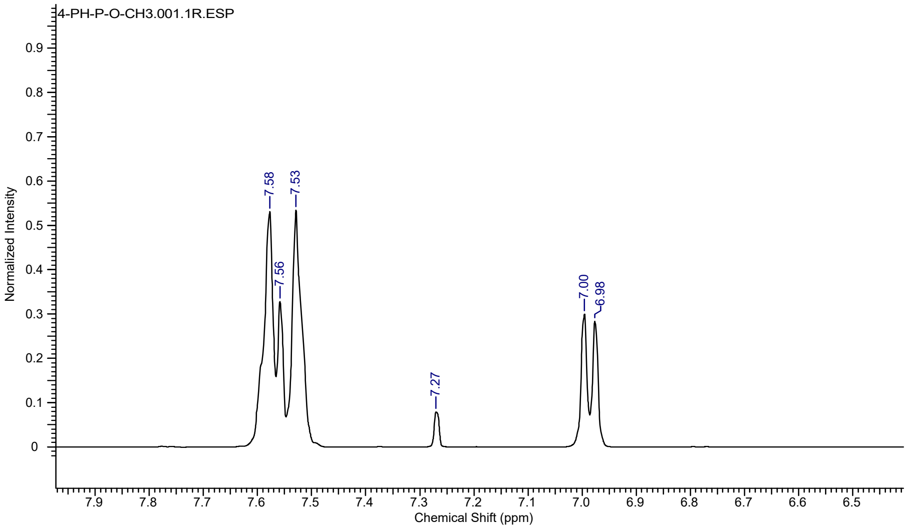

C. ^{13}C NMR

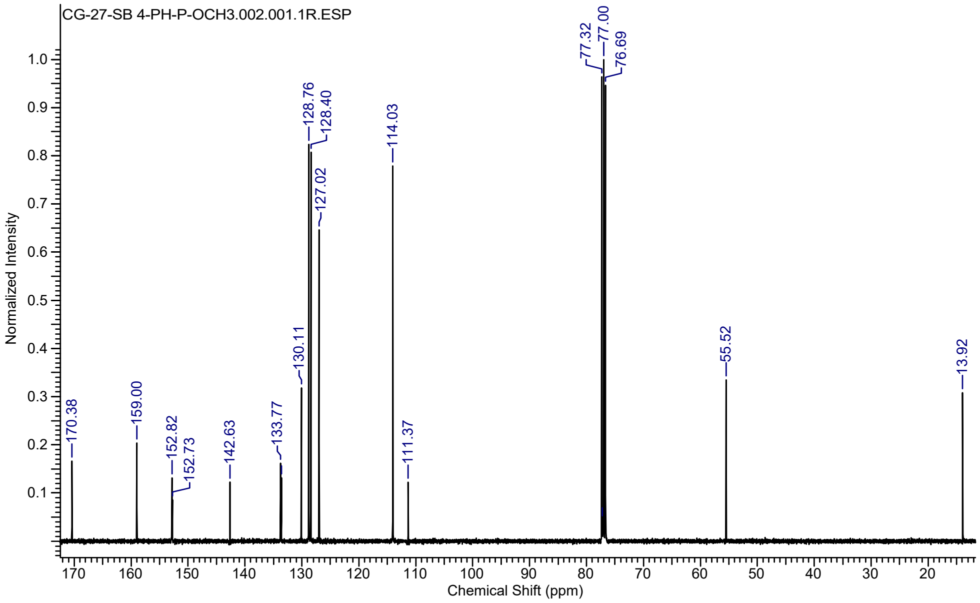

D. ^{13}C NMR zoom

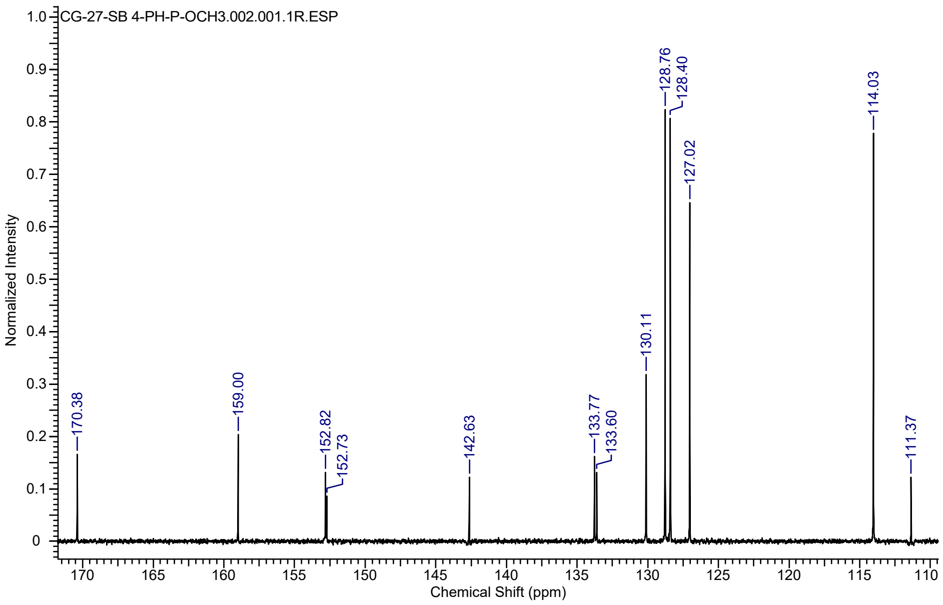

12. ***3-methyl-6-(4-methylphenyl)-4-phenyl-6H,7H-[1,2]oxazolo[3,4-d]pyridazin-7-one, 2l.***

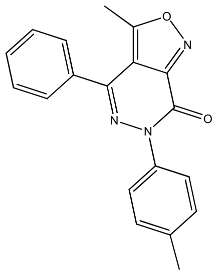

^{1}H NMR (400 MHz, CDCl3): δ 7.60-7.58 (m, J=4 ,2H);7.55-7.53(t, J=4,5H); 2.59 (s, 3H);2.40(s,3H).^{13}C NMR 170.58; 152.75; 133.48; 130.30; 128.87; 128.38; 127.68; 115.82; 115.60; 111.40;13.95 C19H15N3O2 MW: 317.34; HRMS m/z Calc'd for C19H15N3O2 317.3413, Found: 317.1164.

A. ^{1}H NMR

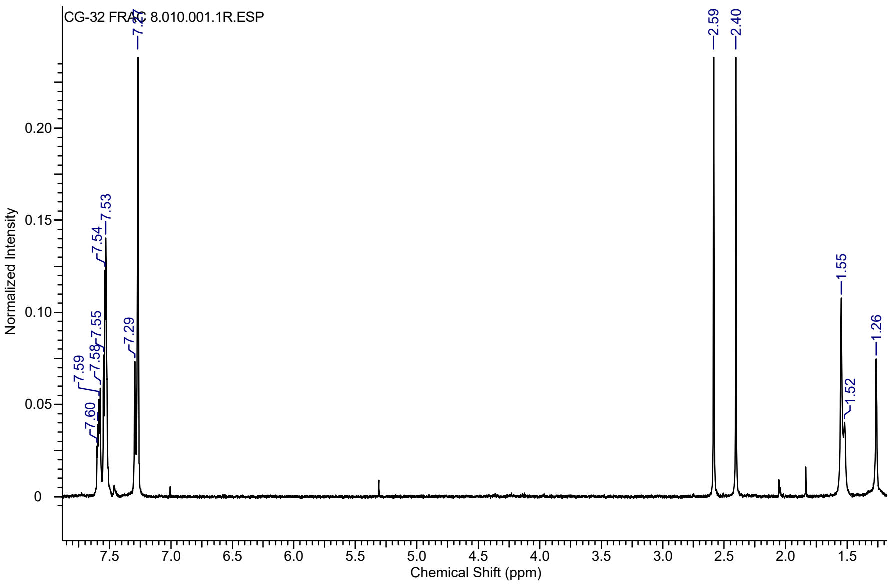

B. ^{1}H NMR Aromatic expanded

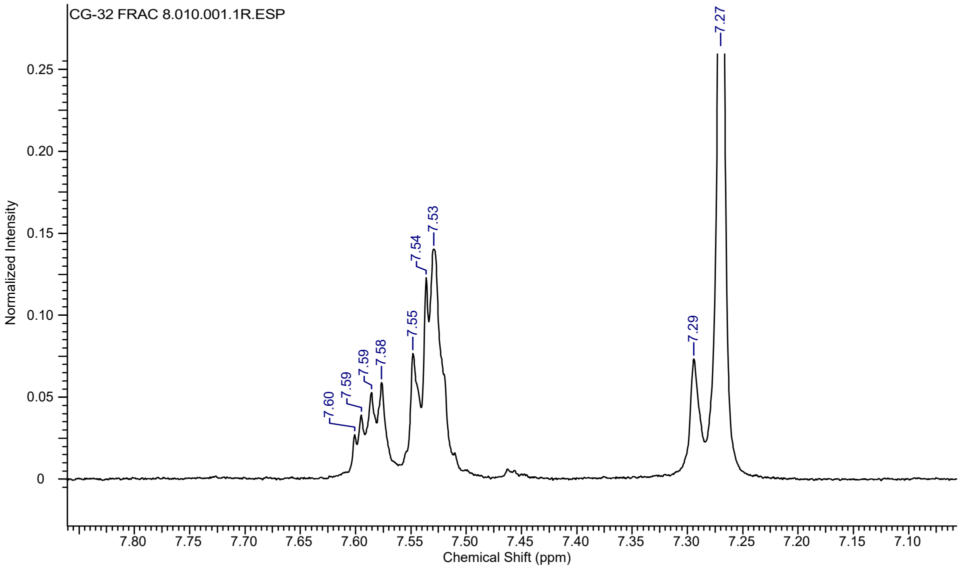

C. ^{13}C NMR

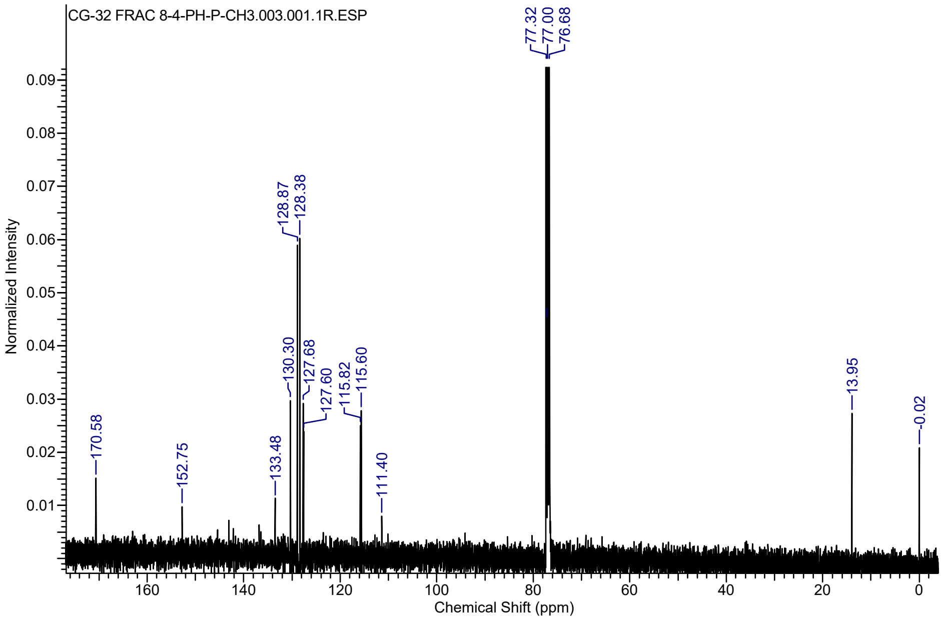

D. ^{13}C NMR zoom

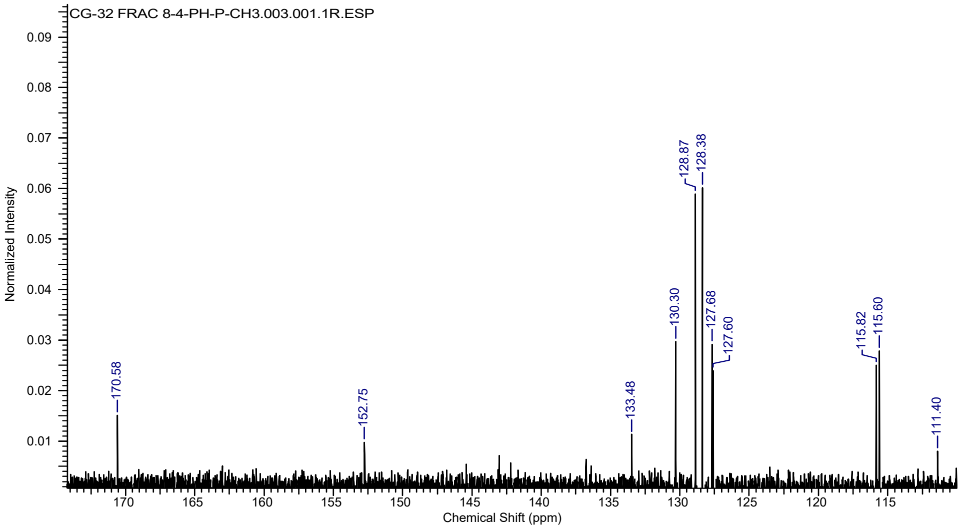

15. 6-[3,5-bis(trifluoromethyl)phenyl]-3-methyl-4-phenyl[1,2]oxazolo[3,4-d]pyridazin-7(6H)-one, .j.

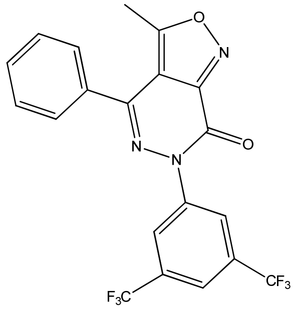

1H NMR (400 MHz, CHLOROFORM-d) d ppm 8.25 - 8.26 (m, 2 H) 7.87 - 7.88 (m, 1 H) 7.59 (s, 5 H) 2.60 - 2.60 (m, 3 H);^{13}C NMR: δ 171.23, 152.78, 152.43, 144.33, 144.91,132.96,132.37,132.03, 130.96, 129.01, 128.35; 125.82, 121.35, 111.29, 13.96. Chemical Formula: C20H11F6N3O2;Exact Mass: 439.0755;Molecular Weight: 439.3107;m/z: 439.0755 (100.0%), 440.0789 (21.6%), 441.0823 (2.2%), 440.0726 (1.1%) Found: 439.08

A. ^{1}H NMR

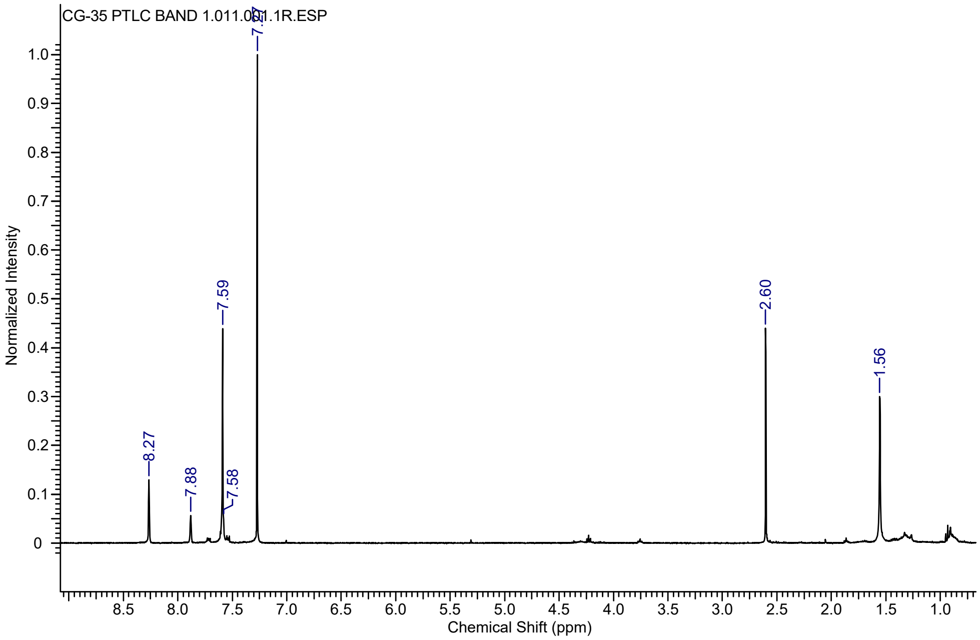

B. ^{1}H NMR Aromatic expanded

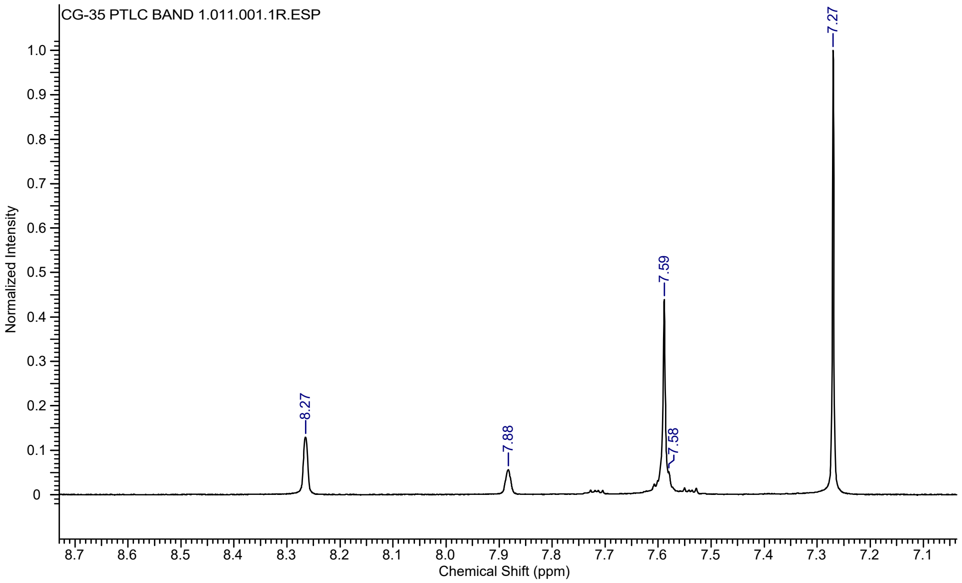

C. ^{13}C NMR

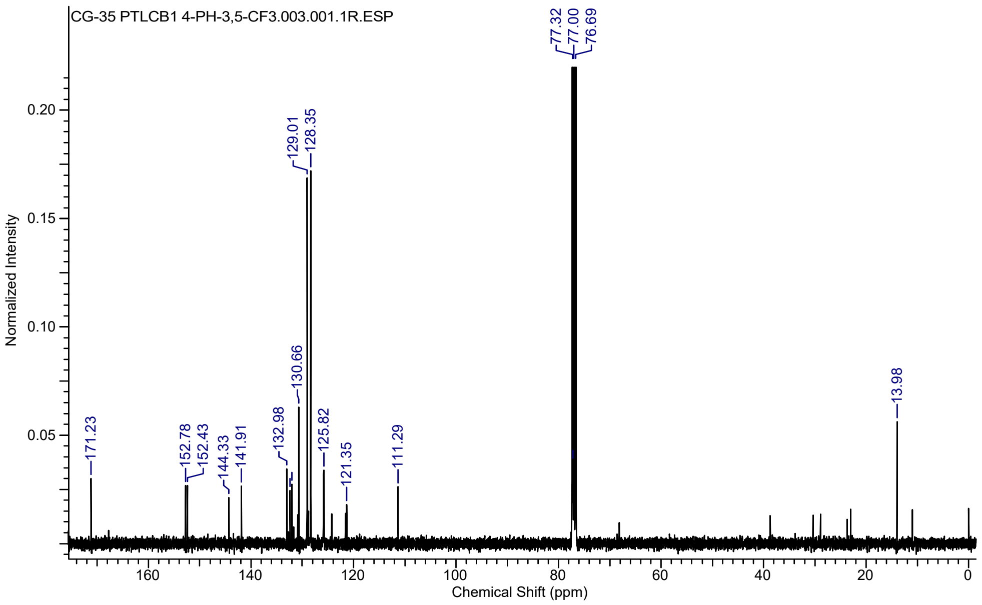

D. ^{13}C NMR zoom

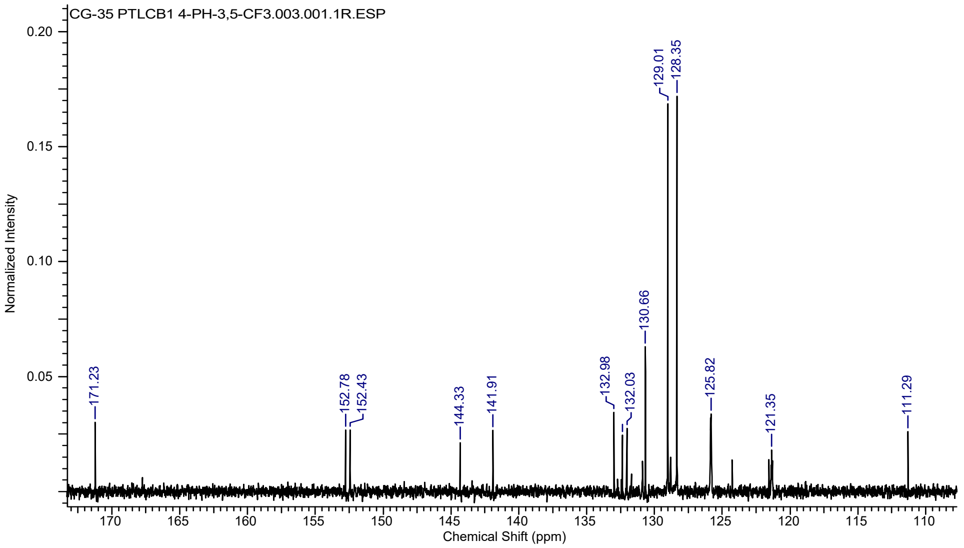

16. 6-(4-fluorophenyl)-3-methyl-4-phenyl[1,2]oxazolo[3,4-*d*]pyridazin-7(6*H*)-one, **2m.**

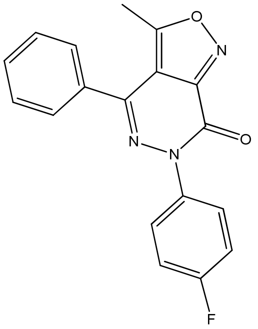

^{1}H NMR (400 MHz, CHLOROFORM-d) d ppm 7.66 (dd, J=7.34, 5.26 Hz, 2H) 7.58 (br. s., 2 H) 7.55 (m, 3 H) 7.17 (t, J=8.01 Hz, 2 H) 2.59 (s, 1 H), ^{13}C NMR: δ 170.29, 152.89, 152.71, 142.62, 138.31,137.96,133.68,130.13, 129.44, 128.78, 128.43; 125.61,111.40, 21.16,13.94, C18H12FN3O2,Exact Mass: 321.0914,Molecular Weight: 321.3052,m/z: 321.0914 (100.0%), 322.0947 (19.5%), 323.0981 (1.8%), 322.0884 (1.1%), Found:322.0970

A. ^{1}H NMR

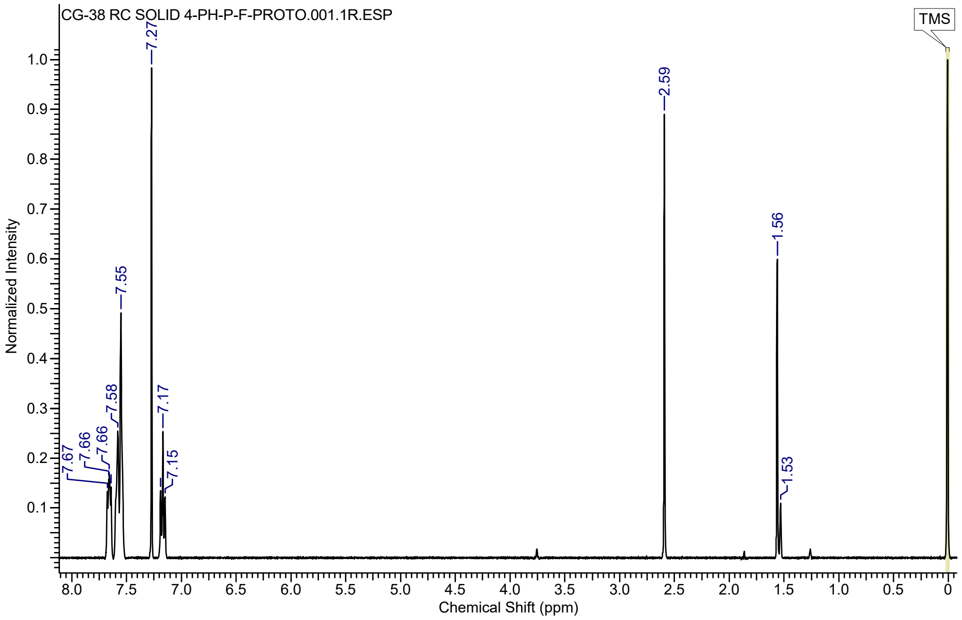

B. ^{1}H NMR Aromatic expanded

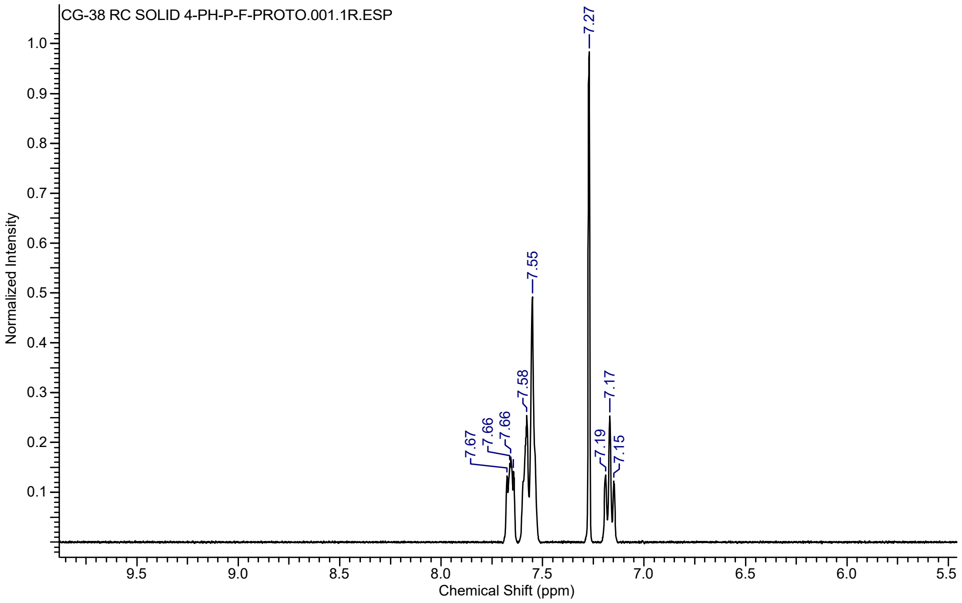

C. ^{13}C NMR

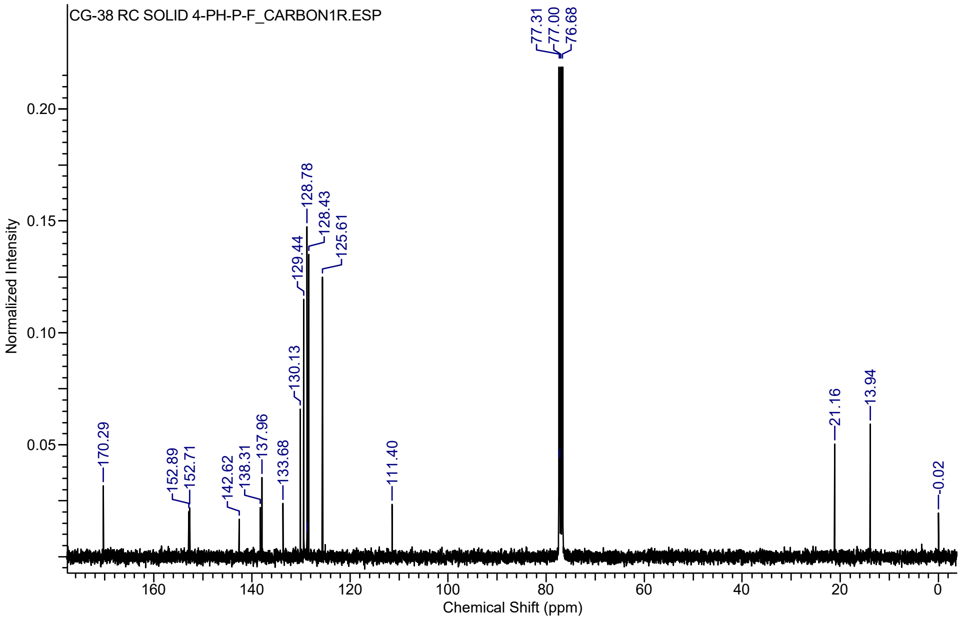

D. ^{13}C NMR zoom

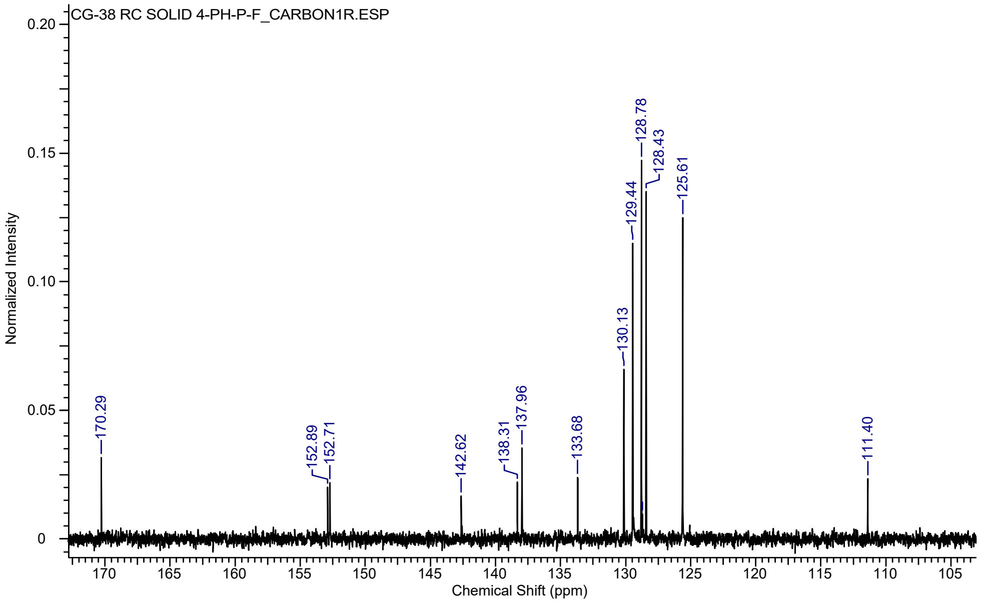

17. 6-(2,4-difluorophenyl)-3-methyl-4-phenyl[1,2]oxazolo[3,4-*d*]pyridazin-7(6*H*)-one, **2n.**

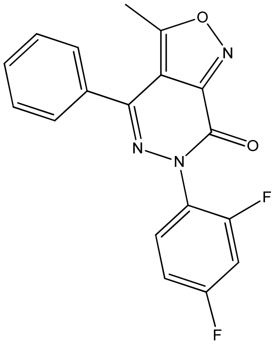

^{1}H NMR (400 MHz, CHLOROFORM-*d*) d ppm 7.54 (br. s., 6 H) 7.01 (t, *J*=8.38 Hz, 2H) 2.58 (s.3 H), ^{13}C NMR: δ 17*0.82*, 164.06, 163.95, 161.45, *158.99,158.87,156.46,156.33,* 1*52.48*,152.27, 1*43.60*, 1*33.21*,130.35,128.88,128.39,124.99,124.86,124.82,111.96,111.74,111.53,105.35,105.12,105.09,104.86, 13.89. C18H11F2N3O2Exact Mass: 339.0819Molecular Weight: 339.2956m/z: 339.0819 (100.0%), 340.0853 (19.5%), 341.0886 (1.8%), 340.0790 (1.1%), Found:340.0895

A. ^{1}H NMR

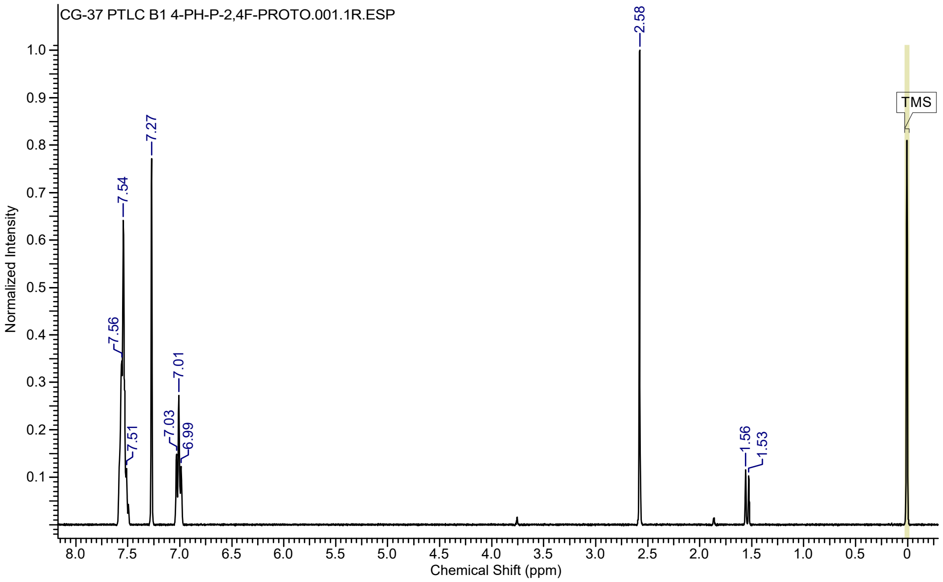

B. ^{1}H NMR Aromatic expanded

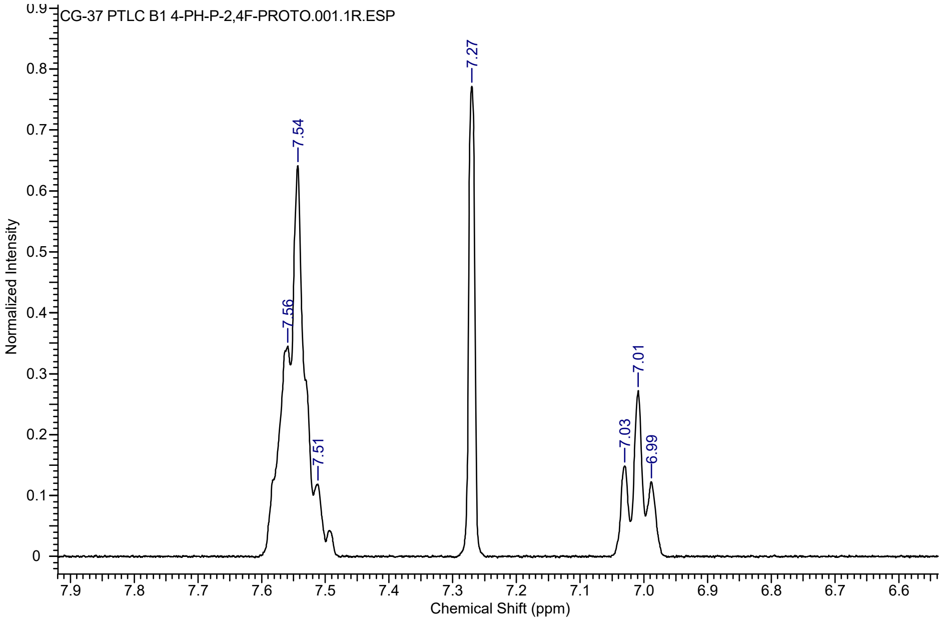

C. ^{13}C NMR

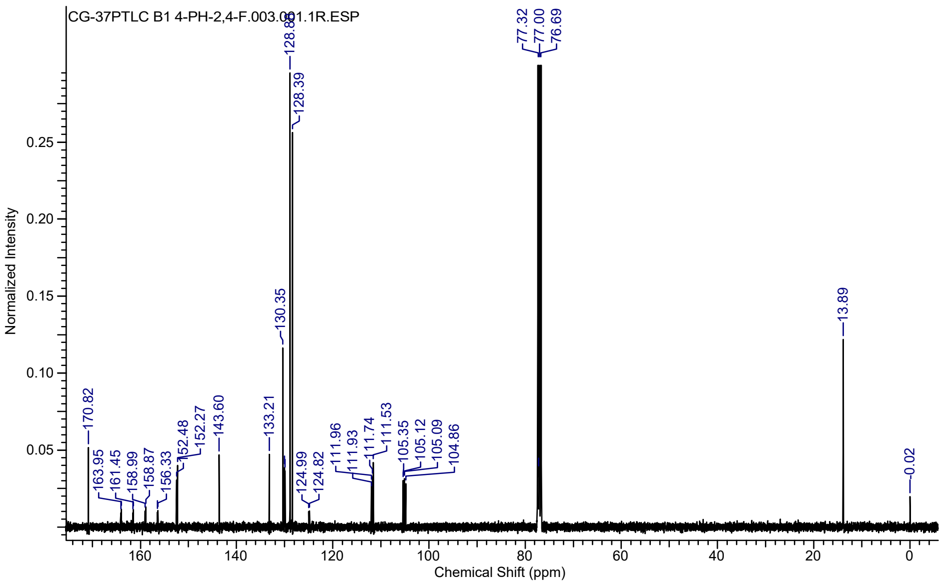

D. ^{13}C NMR zoom

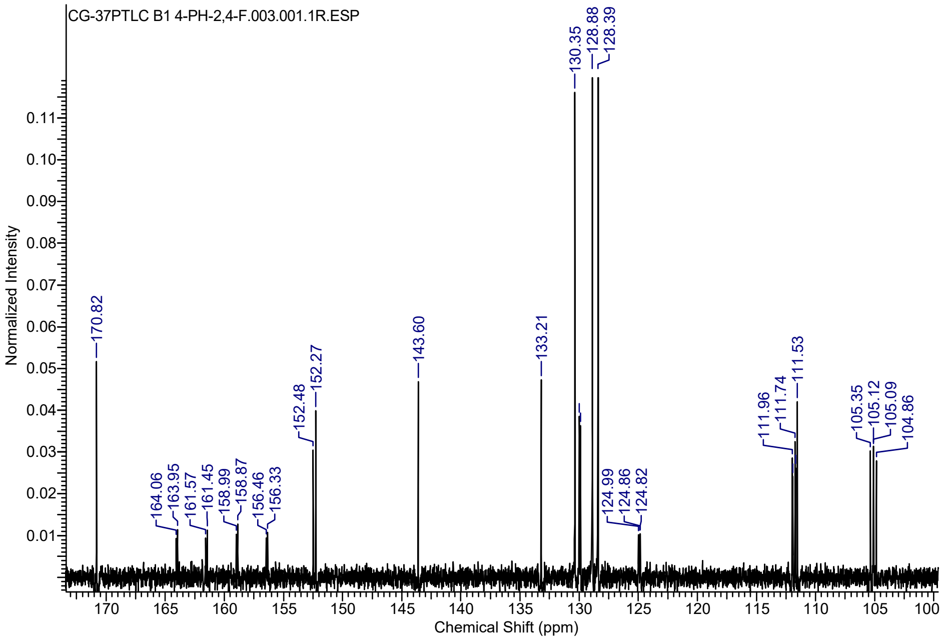

Supplement: Supplementary file 1 [file molecules-28-06800-s001.zip › molecules-2533342-supplementary.pdf]
